# Micro‐Organ Chip Deciphers Tumor‐Derived G‐CSF as Remote Commander of Lung Pre‐Metastatic Niche via VEGFA‐KDR Cascade

**DOI:** 10.1002/advs.202518584

**Published:** 2025-11-22

**Authors:** Jingxin Zhang, Xiaoying Huang, Lingchuan Ma, Zixing Chen, Tianyao Li, Lijian Wang, Yutong Guo, Hu Xu, Junqi Li, Jiang‐Jiang Qin, Xiang Wang, Yuhong Cao, Kai Miao

**Affiliations:** ^1^ Faculty of Health Sciences University of Macau Macau SAR 519000 China; ^2^ CAS Key Laboratory for Biomedical Effects of Nanomaterials and Nanosafety National Center for Nanoscience and Technology Chinese Academy of Sciences Beijing 100190 China; ^3^ University of Chinese Academy of Sciences Beijing 100049 China; ^4^ College of Ecology Lanzhou University Lanzhou 730000 China; ^5^ Clinical Research and Translational Medicine Department The Third Affiliated Hospital of Zhengzhou University Zhengzhou Henan 450000 China; ^6^ Tianjian Laboratory of Advanced Biomedical Sciences Zhengzhou University Zhengzhou Henan 450000 China; ^7^ Center for Innovative Drug Research Hangzhou Institute of Medicine (HIM) Chinese Academy of Sciences Hangzhou Zhejiang 310018 China; ^8^ Department of Medical Oncology Peking Union Medical College Hospital Chinese Academy of Medical Sciences and Peking Union Medical College Beijing 100032 China; ^9^ MOE Frontier Science Centre for Precision Oncology University of Macau Macau SAR 519000 China; ^10^ Zhuhai UM Science & Technology Research Institute Hengqin 519031 China

**Keywords:** angiogenesis, G‐CSF, KDR, micro‐organ chip, pre‐metastatic niche

## Abstract

Metastasis is the leading cause of cancer‐related mortality. During metastatic progression, distant organs form a pre‐metastatic niche (PMN), creating a permissive microenvironment that facilitates circulating tumor cell (CTC) colonization. To investigate pulmonary PMN formation in breast cancer, a micro‐organ chip is employed that enables contact‐independent coculture of tumor and lung tissues. This model reveals that PMN formation is governed by tumor‐secreted factors without requiring direct tumor cell contact and exhibits non‐tumor‐type specificity. It is found that coculture with tumor tissue upregulates vascular endothelial growth factor (VEGF) receptor‐2 (VEGFR2/KDR) in lung capillary cells. Through integrated single‐cell RNA sequencing and cytokine array analysis, granulocyte colony stimulating factor (G‐CSF) is identified as a key tumor‐derived mediator that modulates the pre‐metastatic niche through activating the VEGFA‐KDR signaling axis in the lung, thereby promoting angiogenesis and PMN development. This study highlights the G‐CSF‐KDR axis as a potential therapeutic target for inhibiting breast cancer metastasis.

## Graphical abstract

Breast cancer reprograms the lung into a receptive pre‐metastatic niche via a novel G‐CSF–VEGFA–KDR signaling axis. Using a micro‐organ chip that enables contact‐independent coculture, this study uncovers how tumor‐secreted G‐CSF activates lung capillary KDR to drive angiogenesis and prime the soil for metastasis—without direct tumor contact. These findings reveal the G‐CSF–KDR axis as a promising target for blocking metastatic spread.

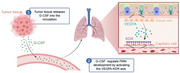

## 1 Introduction

Metastasis is the predominant cause of cancer‐related mortality, accounting for over 90% of cancer deaths. In breast cancer, prognosis varies dramatically—patients with localized tumors exhibit a 91.7% 5‐year survival rate, whereas those with distant metastases face a precipitous decline to 32.6%.^[^ ^1^ ^]^ This stark disparity underscores the urgent need to target metastatic progression. The lungs represent a major metastatic site in breast cancer, accounting for 21–32%^[^ ^2^ ^]^ of distant metastases, with pulmonary metastases conferring particularly poor outcomes (median survival: 25 months).^[^ ^3^ ^]^

The establishment of pre‐metastatic niches (PMNs)—immunosuppressive microenvironments that support disseminated tumor cell colonization—is now recognized as a critical prerequisite for metastasis. Stephen Paget's “seed and soil” hypothesis^[^ ^4^ ^]^ posits that circulating tumor cells (CTCs, “seeds”) require receptive host tissues (“soil”) to colonize.^[^ ^5^ ^]^ This concept was mechanistically validated when Kaplan et al. identified bone marrow‐derived VEGFR1+ hematopoietic progenitor cells (HPCs) as early architects of PMNs.^[^ ^6^ ^]^ Subsequent studies revealed that PMN formation involves various crosstalk between tumor‐derived factors^[^ ^7^ ^]^ (e.g., VEGF,^[^ ^8^ ^]^ PIGF,^[^ ^9^ ^]^ TGF‐β^[^ ^10^ ^]^) and host stromal components. Key mediators include: Bone marrow‐derived cells (BMDCs)^[^ ^11^ ^]^ comprise myeloid lineage cells (including macrophages^[^ ^12^, ^13^ ^]^ and neutrophils),^[^ ^14^ ^]^ immunosuppressive populations (such as myeloid‐derived suppressor cells (MDSCs),^[^ ^15^ ^]^ regulatory T cells (Tregs)^[^ ^11^ ^]^), and hematopoietic progenitor cells (HPCs), that collectively participate in immune microenvironment remodeling. Tumor‐derived extracellular vesicles (EVs)^[^ ^16^ ^]^ mediate intercellular communication by delivering small nuclear RNAs (snRNAs)^[^ ^17^ ^]^ and microRNAs (miRNAs)^[^ ^18^ ^]^ to reprogram alveolar type 2 cells toward immunosuppressive phenotypes.^[^ ^19^ ^]^ Concurrently, stromal reprogramming occurs through fibroblast‐ and endothelial‐derived factors (IL‐10, SDF‐1α,^[^ ^20^ ^]^ and fibrinogen^[^ ^21^ ^]^), which collectively promote immune evasion, extracellular matrix (ECM) remodeling, and angiogenesis.^[^ ^22^ ^]^ Gui et al. demonstrated that tumor‐derived factors activate p38α in lung fibroblasts, driving pre‐metastatic niche formation via FAP‐mediated ECM remodeling and neutrophil recruitment.^[^ ^23^ ^]^

Despite these advances, fundamental questions remain elusive regarding pulmonary PMN formation in breast cancer: 1) Contact‐dependence: Is physical tumor cells and host interaction necessary for PMN initiation. 2) Dominant regulators: what are the mediators govern PMN maturation and functional specialization.

To address these questions, we utilized a micro‐organ chip that enables tumor–lung coculture while maintaining physical segregation. Through integrated single‐cell RNA sequencing and cytokine profiling, we identified granulocyte colony‐stimulating factor (G‐CSF) as a master regulator that hijacks the VEGFA‐KDR axis in capillary (CAP) cells, driving PMN maturation through angiogenic specialization (CD31^+^EMCN^+^ microvascular expansion), and ECM remodeling (fibronectin/vimentin deposition). These findings shift the therapeutic paradigm toward proactive PMN disruption and establish the G‐CSF‐VEGFA‐KDR axis as a promising therapeutic target for metastasis prevention in breast cancer.

## 2 Results

### 2.1 Establishment of an Ex Vivo Pulmonary PMN Model

**Figure 1:**
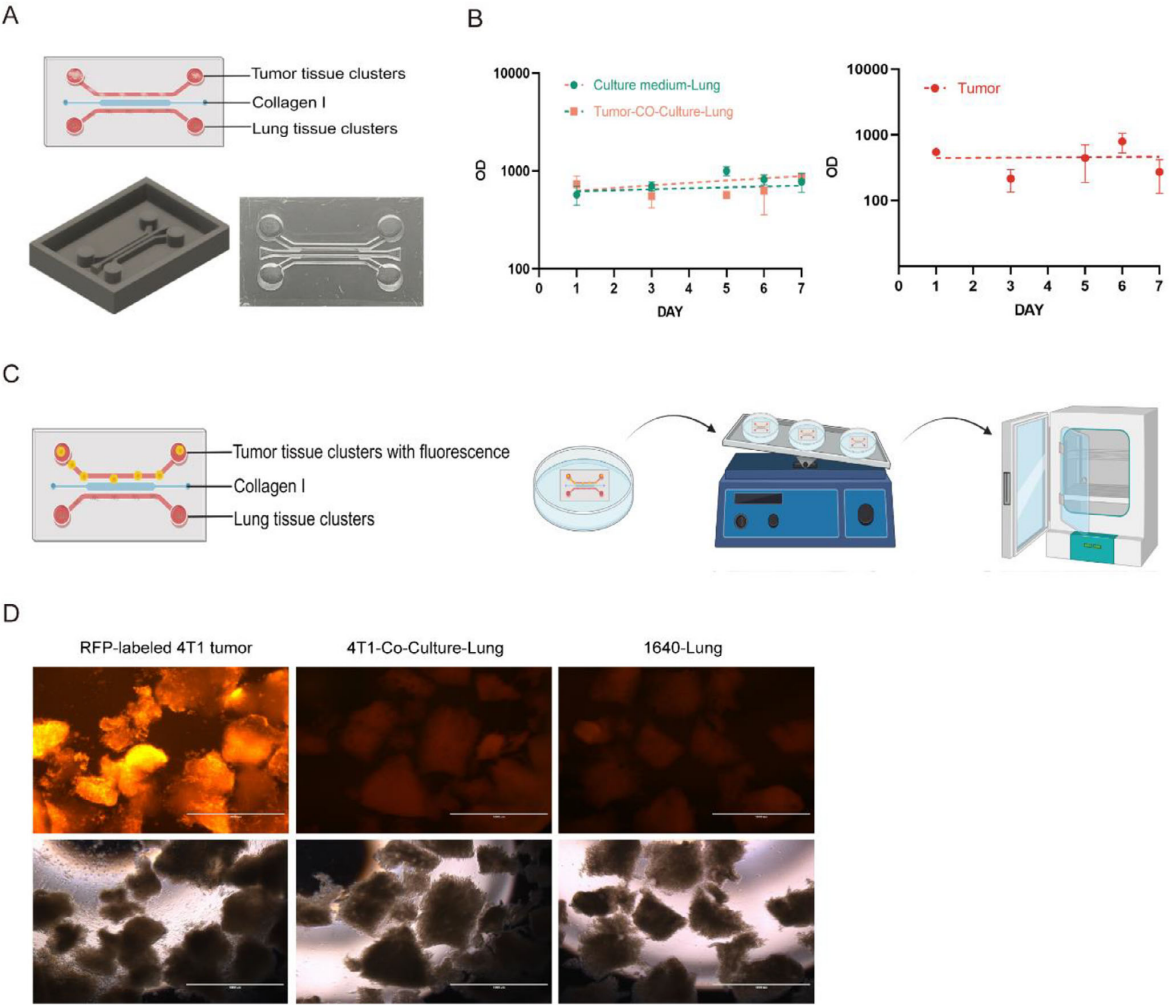
Design and feasibility of micro‐organ chip for investigating breast cancer lung PMN formation. A) Micro‐organ chip architecture (Top), additive manufacturing of the micro‐organ chip mold via 3D printing (Lower left), and PDMS replica post‐demolding (Lower right). B) The OD value of different treated lung tissue clusters and tumor tissue clusters from one day to seven days (n=3). C) Workflow for determining the formation of collagen barrier layers in the middle chamber. D) Fluorescence imaging of 4T1‐RFP diffusion between adjacent tissue chambers (Day 6 post‐barrier maturation, Scale bars, 1000 µm).

To overcome the limitations of existing models in studying pre‐metastatic niche (PMN) dynamics, we engineered a 3D micro‐organ chip that recapitulates the spatiotemporal evolution of lung PMN formation during breast cancer metastasis. This microphysiological system employs a polydimethylsiloxane (PDMS)‐based microfluidic device (Figure S1A, Supporting Information) featuring three functionally independent microchannels: 1) an upper tumor tissue chamber, 2) a lower lung tissue channel, and 3) a thermo‐responsive collagen barrier (37 °C gelation) that permits robust cytokine diffusion while effectively restricting cellular migration, as validated by our cytokine diffusion assay (Figure S1B, Supporting Information). This tripartite structure enables real‐time monitoring of tumor–lung crosstalk without direct cell contact – a critical requirement for PMN mechanistic studies (**Figure** **1**A; Figure S1A, Supporting Information).

To validate tissue viability in the micro‐organ chip system, we performed longitudinal monitoring of tissue fragments under physiological culture conditions (37 °C, 5% CO_2_, 20 rpm orbital agitation). AlamarBlue assays on tumor clusters over 7 days (Figure 1B; Figure S1C, Supporting Information) confirming sustained viability, confirming the PDMS‐based system's ability to maintain physiological tissue homeostasis.

Compartmental integrity was further verified using RFP‐labeled 4T1 mammary tumors (orthotopically derived from BALB/c mice) cocultured with syngeneic lung tissues across the collagen barrier (Figure 1C). Six‐day continuous monitoring (Figure 1D; Figure S1D, Supporting Information) revealed complete absence of red fluorescence signal in lung compartments, establishing the system's capacity for effective physical segregation.

This system bridges the resolution gap between reductionist in vitro models and in vivo complexity, enabling analysis of PMN‐initiating events ‐ from early vascular remodeling to metastatic colonization.

### 2.2 PMN Formation Exhibits Tumor Individual Independent Characteristics

Lung pre‐metastatic niche formation is characterized by defined molecular alterations, including extracellular matrix remodeling (ECM) and angiogenesis.^[^ ^24^, ^25^ ^]^ To systematically investigate whether tumor educated lungs develop functional PMNs, we leveraged our micro‐organ chip to coculture tissues and assessed key hallmarks: tumor cell colonization, vascular remodeling (via CD31 and Endomucin/EMCN) and ECM reprogramming (via Fibronectin and Vimentin), which are critical for creating a pro‐metastatic microenvironment.

**Figure 2:**
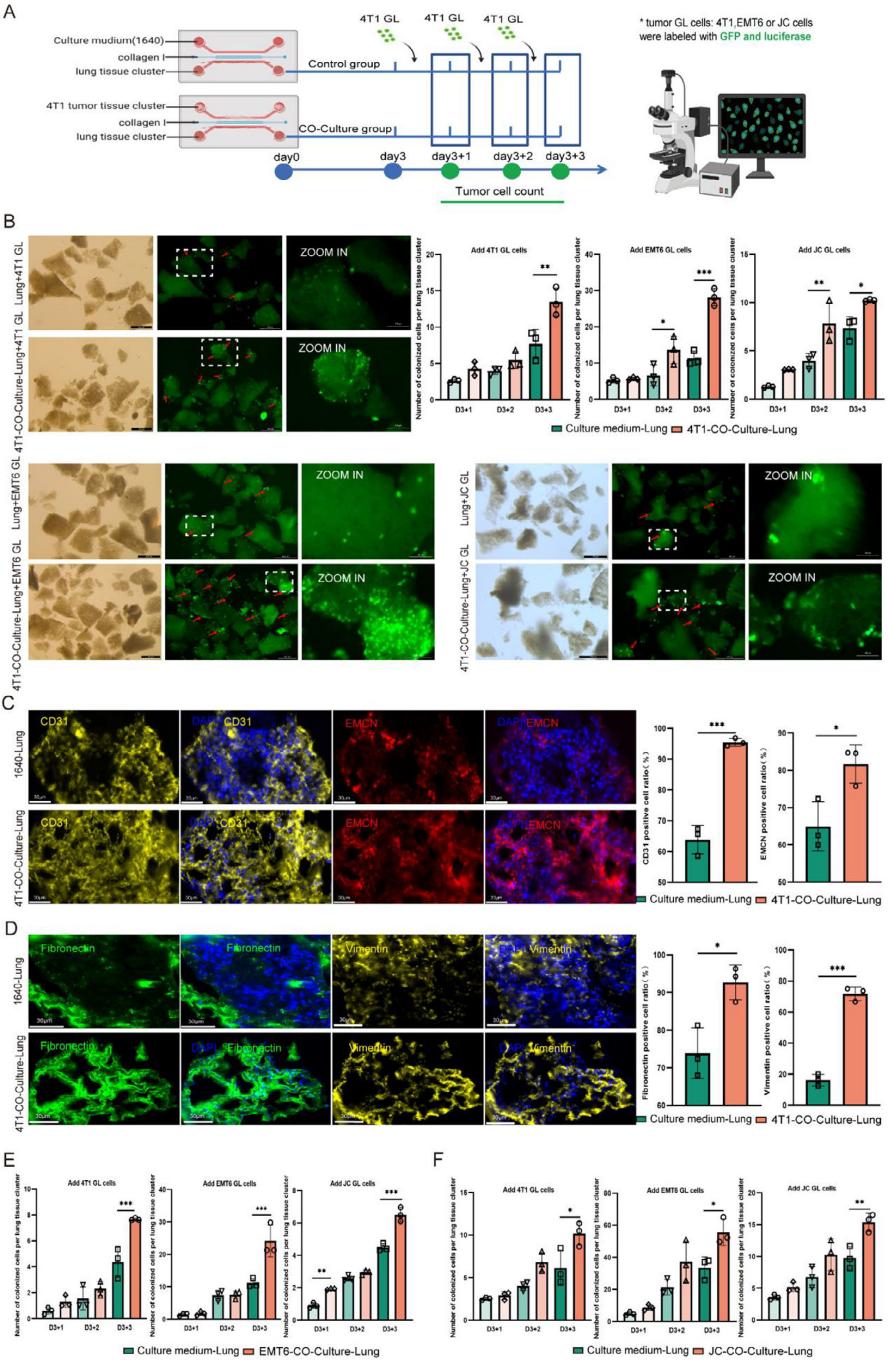
Characterization of pulmonary PMN formation using micro‐organ chip. A) Schematic workflow for PMN induction and tumor cell colonization assays. Lung tissues were harvested for tumor cell colonization analysis after 3 days of co‐culture plus 1–3 days tumor cell seeding. B) **Top‐left**: Representative fluorescence images showing 4T1‐GFP+ cell colonization in pulmonary tissue clusters. **Top‐right**: Normalized colonization density (colonized cells/lung clusters) of GFP positive 4T1, EMT6, and JC cells in 4T1 tumor tissue cluster‐co‐cultured lung tissues versus control lung tissues. **Bottom**: Representative fluorescence images showing EMT6‐GFP+ and JC‐GFP+ cell colonization in 4T1‐tumor tissue co‐cultured pulmonary tissue clusters (Scale bars: 500 µm; n=3 biological replicates; NS: not significant; *p < 0.05, **p < 0.01, ***p < 0.001 by one‐way ANOVA). C) **Left**: Immunofluorescence analysis of PMN‐associated remodeling in micro‐organ chip cultured lung tissues. Nuclear staining (DAPI, blue), Vascular remodeling (CD31+, yellow, EMCN, red). (Scale bars: 30 µm). **Right**: Quantitative analysis of CD31+, EMCN+ deposition. (n=3; *p < 0.05, **p < 0.01, ***p < 0.001 by two‐tailed t‐test). D) **Left**: Immunofluorescence analysis of PMN‐associated remodeling in micro‐organ chip cultured lung tissues. Nuclear staining (DAPI, blue), ECM reorganization (Fibronectin, green, Vimentin, yellow). (Scale bars: 30 µm). **Right**: Quantitative analysis of Fibronectin and Vimentin deposition. (n=3; *p < 0.05, **p < 0.01, ***p < 0.001 by two‐tailed t‐test). E) Tumor subtype‐crossing colonization assay. Normalized colonization value (colonized cells/lung clusters) of 4T1‐GL, EMT6‐GL, and JC‐GL cell colonization in lung tissues pre‐conditioned with EMT6 tumor tissues. (n=3; NS: not significant; *p < 0.05, **p < 0.01, ***p < 0.001 by one‐way ANOVA). F) Normalized colonization density (colonized cells/lung clusters) of 4T1‐GL, EMT6‐GL, and JC‐GL cell colonization in lung tissues pre‐conditioned with JC tumor tissues. (n=3; NS: not significant; *p < 0.05, **p < 0.01, ***p < 0.001 by one‐way ANOVA).

Beginning on day 3 and daily thereafter, we introduced 1 × 10^3^ GFP‐luciferase‐labeled tumor cells (4T1‐GL) into the lung channel to simulate circulating tumor cell (CTC) dissemination. Tumor cell adhesion was quantified after triple PBS washes performed daily starting from day 3 (**Figure** **2**A). Strikingly, tumor‐co‐cultured lungs exhibited a 74.80% increase in 4T1‐GL adhesion at day 6 (Figure 2B), demonstrating successful recapitulation of metastasis‐permissive microenvironments in vitro.

Using immunofluorescence staining, we further characterized hallmark features of lung PMN after tumor coculture. As shown in Figure 2C, both CD31 (Platelet endothelial cell adhesion molecule‐1, PECAM‐1/CD31, a pan‐endothelial marker)^[^ ^26^ ^]^ and EMCN (a specific marker of pulmonary capillary endothelial cells)^[^ ^27^ ^]^ positive areas are significantly increased (49.55% increase in CD31 vessels, and 46.61% elevation in EMCN+ capillaries), indicating a substantial vascular remodeling (Figure 2C). Further analysis on ECM reprogramming demonstrates an increased fibronectin deposition (from 73.89% to 92.72%) and vimentin accumulation (from 16.17% to 71.77%) (Figure 2D). Remarkably, physical segregation in our micro‐organ chip confirmed that without direct cell contact that may induced by soluble tumor‐derived factors alone are sufficient to induce full PMN maturation.

Cross‐seeding experiments exhibit 4T1 co‐cultured lungs significantly enhanced the adhesion of different cancer individuals, with around two‐fold increase (11.33% vs 28.16%) for EMT6 and 39.05% increase (7.35% vs 10.22%) for JC tumors (Figure 2B). Conversely, we observed that lungs educated by either EMT6 or JC tumors, could also increase different tumor cell colonization (Figure 2E,F; Figure S2A,B, Supporting Information), underscoring the universality of PMN receptivity.

These findings collectively establish fundamental principles: 1) Breast tumors remotely engineer a “metastasis‐permissive soil” predominantly through secretory components independent of direct physical interactions; 2), PMN receptivity constitutes a universal property that transcends intrinsic tumor individuals, enabling metastatic colonization.

### 2.3 Single‐Cell Sequencing Identifies CAP Cell Derived KDR is the Key Driver in PMN Formation

**Figure 3:**
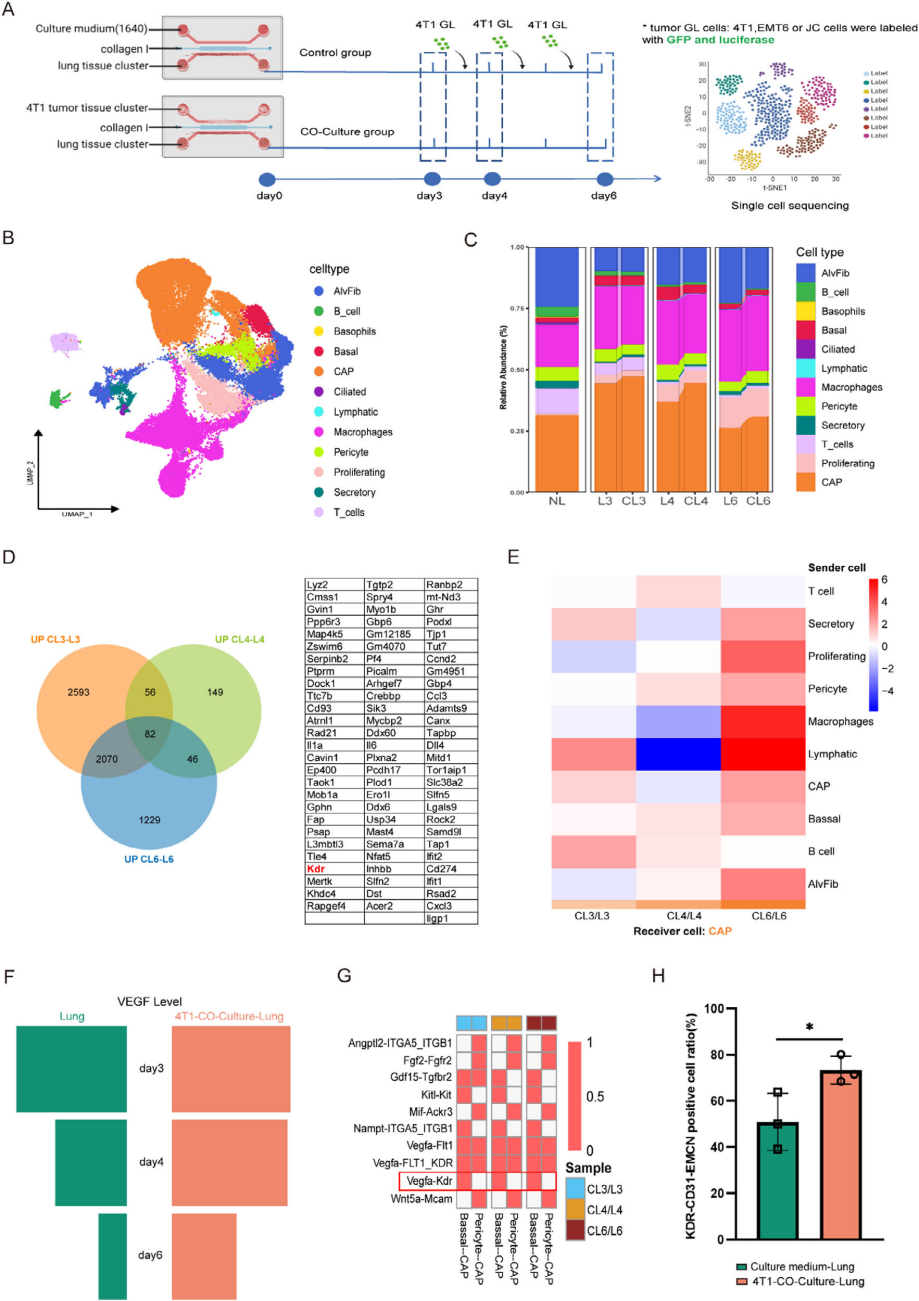
Single‐cell RNA sequencing analysis identifies key cellular populations and master molecular in PMN formation. A) Schematic workflow of single‐cell RNA‐seq sample processing and bioinformatic analysis. On day 0, negative control lung tissue samples were collected. Subsequently, tumor co‐culture lung (CL) and control lung tissues (L) were harvested at days 3, 4, and 6, yielding a total of 7 lung tissue specimens. Following single‐cell dissociation and erythrocyte depletion, all samples underwent single‐cell RNA sequencing. B) UMAP projection of 12 distinct cell clusters identified in lung tissues. C) Dynamic changes in cellular composition in tumor‐co‐cultured lung tissues (CL) versus controls (L) across time points (Time points are designated as N, corresponding to day 3 (N=3), day 4 (N=4), or day 6 (N=6) post‐intervention). D) **Left**: Venn diagram analysis of upregulated differentially expressed genes (DEGs) in CAP cells. Orange, green and blue circles represent up‐regulated DEGs across CL3/L3, CL4/L4, and CL6/L6 conditions, respectively. **Right**: A list of 82 DEGs demonstrating consistent upregulation across all time points. E) The heatmap demonstrates that basal cells and B cells collectively exhibit progressively enhanced signaling to CAP cells (the recipient population) across different culture time points. F) The butterfly plot demonstrates that VEGF signaling from basal cells shows progressively enhanced secretion in tumor co‐cultured lung tissues compared to control lung tissues with prolonged culture duration. G) The heatmap displays coordinated temporal patterns of ligand–receptor interactions targeting CAP cells from BASAL cells or pericytes across different culture time points. H) Quantification of KDR+ cells among CAP cells (n=3; mean ± SEM; *p < 0.05, **p < 0.01, ***p < 0.001 by two‐tailed t‐test).

To dissect the cellular dynamics underlying PMN establishment, we performed single‐cell RNA sequencing (scRNA‐seq) on 92042 high‐quality cells isolated from seven lung tissue cohorts, including naive controls and tumor or medium co‐cultured lung samples at days 3, 4, and 6 (**Figure** **3**A). Unsupervised clustering identified 12 transcriptionally distinct cell populations (Figure 3B; Figure S3A, Supporting Information), among which capillary (CAP) cells exhibiting progressive expansion during PMN maturation (Figure 3C). Tumor co‐cultured lung tissue induced significantly increases in CAP cell proportions, i.e., from 44.53% to 47.32%, from 37.00% to 44.66% and from26.42% to 30.84% at days 3, 4, and 6, respectively compared to controls (Figure 3C; Figure S3B, Supporting Information). As demonstrated by immunofluorescence staining in Figure 2C, the vascular and microvascular networks were markedly expanded in tumor co‐cultured lung tissue clusters—representing a structural hallmark of CAP cells activation. This temporal correlation strongly suggested a central role for CAP cells in orchestrating PMN formation.

Differential expression gene (DEG) analysis of CAP cells revealed 82 consistently upregulated genes in tumor‐co‐cultured lungs, with no genes showing consistent downregulation (Figure 3D; Figure S3C, Supporting Information). Gene Ontology (GO) enrichment analysis revealed these genes were significantly associated with critical biological processes including (Figure S3E, Supporting Information): Biological Processes: cell proliferation (GO:0008283), angiogenesis (GO:0001525), cell adhesion (GO:0045785), and epithelial‐mesenchymal transition (EMT, GO:0001837) (p<0.01, FDR<0.05); Molecular Functions: signal receptor binding (GO:0005102), cytoskeletal protein binding (GO:0008092), and ATP binding (GO:0005524) functions; and Cellular Compartments: cell junctions (GO:0030054), anchoring junctions (GO:0070161), and actin cytoskeleton (GO:0015629). The results support their role in vascular niche specialization.

Cell–cell interaction profiling demonstrated enhanced communication between basal cells and CAP cells across all timepoints (Figure 3E; Figure S3D, Supporting Information). Specifically, basal cell exhibited increased VEGFA expression during co‐culture (Figure 3F; Figure S4A, Supporting Information), while receptor‐ligand pairing analysis confirmed strengthened VEGFA‐KDR interactions among CAP cells and basal cells (Figure 3G; Figure S4B, Supporting Information) indicating its potential in regulating PMN formation. To further validate it, IF staining exhibiting the increased KDR expression was co‐localized with CD31+EMCN+ positive cells (rising from 50.96% to 73.31%; Figure 3H; Figure S4C, Supporting Information).

Collectively, these results suggested a regulation paradigm wherein tumor‐secreted factors remotely activate the VEGFA‐KDR signaling axis in lung CAP cells, driving PMN maturation through coordinated vascular specialization and stromal remodeling–positioning KDR as the master molecular switch in metastatic niche programming.

### 2.4 KDR Inhibition Disrupts Pulmonary PMN Formation

**Figure 4:**
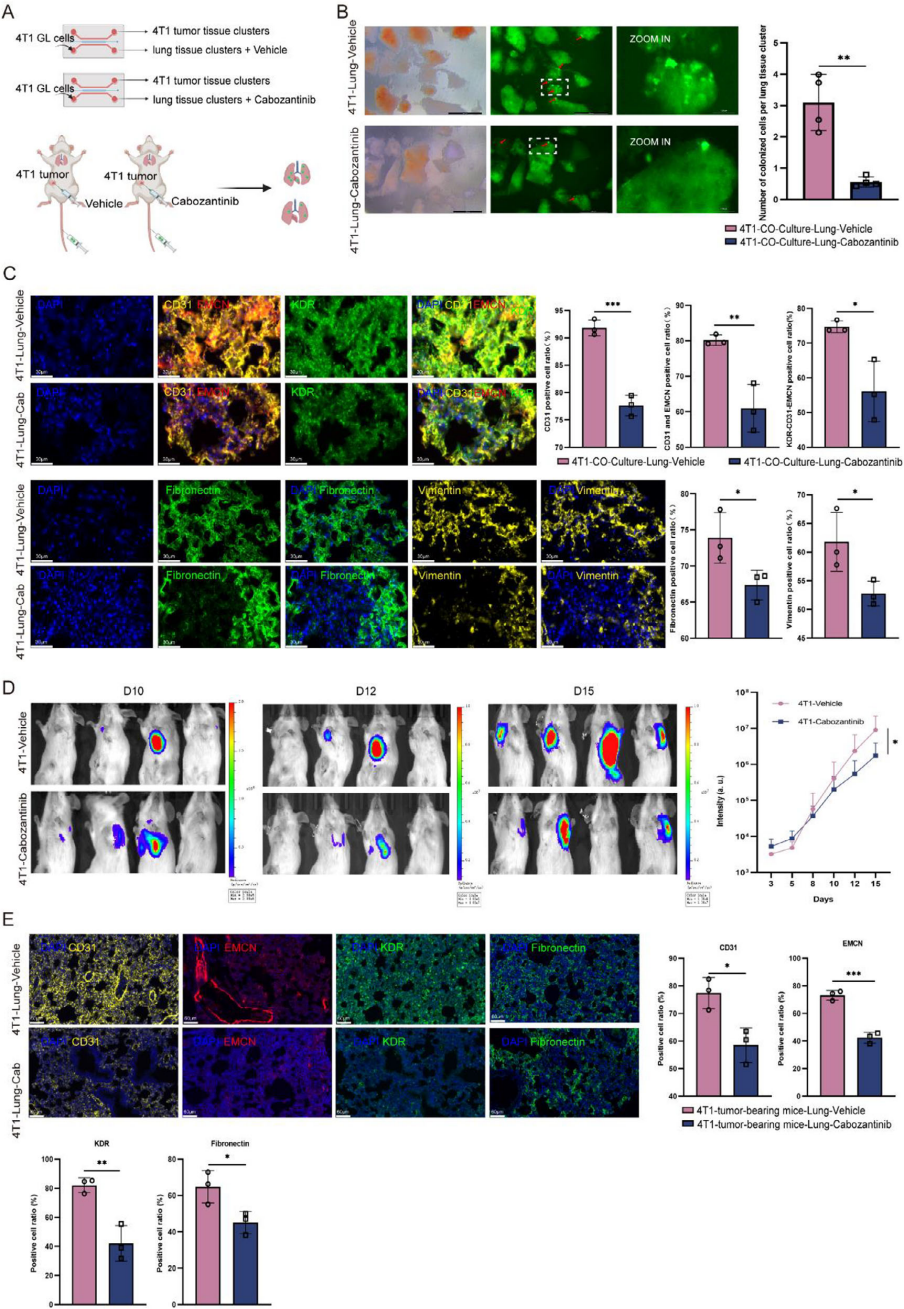
Pharmacological inhibition of KDR disrupts PMN formation in vitro and in vivo. A) Schematic of assessing KDR inhibitor effect in micro‐organ chip in vitro co‐culture system **(Top)** and orthotopic 4T1 breast cancer in vivo model **(Bottom)**. B) **Left**: Representative fluorescence images showing 4T1‐GFP+ cell colonization in tumor‐co‐cultured lung tissue clusters after 3 days continuous treatment with either Cabozantinib or vehicle control. **Right**: Normalized colonization density (cells per clusters) in KDR inhibitor‐ versus vehicle‐treated groups. (Scale bars: 500 µm; *p < 0.05, **p < 0.01, ***p < 0.001 by two‐tailed t‐test). C) **Left**: Immunofluorescence analysis of PMN markers in micro‐organ chip cultured lung tissues. Nuclear staining (DAPI, blue), Vascular remodeling (CD31+, yellow, EMCN, red), KDR (green), ECM remodeling (Fibronectin, green; Vimentin, yellow). (Scale bars: 30 µm). **Right**: Quantitative analysis of CD31+, CD31+ and EMCN+, KDR+, Fibronectin, and Vimentin deposition. (n=3; *p < 0.05, **p < 0.01, ***p < 0.001 by two‐tailed t‐test). D) Representative longitudinal bioluminescence imaging (foci 10–15 days post 4T1‐GL injection) and quantitative fluorescence analysis of pulmonary metastases at 3–15 days post‐4T1‐GL cells injection.  (n=4, *p* = 0.0140, Fisher's LSD post hoc test). E) IF analysis of lung tissues from orthotopic tumor‐bearing mice. Representative images of CD31 and EMCN (angiogenesis), KDR (target engagement), and Fibronectin (ECM remodeling) in lung sections and quantification results. (Scale bars: 60 µm n = 3 mice/group; *p < 0.05, **p < 0.01, ***p < 0.001 by two‐tailed t‐test).

To validate the master role of KDR in PMN establishment, we utilized cabozantinib (XL184), a commonly used VEGFR2 (KDR) inhibitor, in micro‐organ chip models and a mouse orthotopic tumor model (**Figure** **4**A). Tumor‐educated lung tissues were treated with either cabozantinib or vehicle control, followed by 4T1‐GL cells infusion to simulate CTC dissemination. Quantitative analysis revealed a 4.5‐fold reduction in tumor colonization within inhibitor‐treated lungs (Figure 4B), demonstrating that KDR blockade functionally cripples the PMN's capacity to support metastatic seeding.

Multiplex immunofluorescence staining revealed coordinated disintegration across PMN compartments under KDR inhibition (Figure 4C; Figure S5A, Supporting Information). The vascular compartment showed a 14.20–24.04% reduction with indicated by CD31+ area (from 91.83% to 71.63%) and EMCN+ microvessel area (from 80.21% to 60.93%). Concurrently, ECM remodeling was significantly attenuated from 73.89% to 67.37% with fibronectin depletion and 14.58% vimentin suppression (from 61.80% to 52.79%) (Figure 4C). These results collectively established KDR as the central druggable hub orchestrating PMN maturation.

Complementary in vivo studies using orthotopic 4T1 breast cancer models robustly reinforced these findings (Figure 4A). Cabozantinib treatment initiated at day 7 post tumor implantation, followed by intravenous injection of 4T1‐GL cells at day 10 to model pulmonary metastasis. Longitudinal bioluminescence imaging revealed a significant reduction of lung colonization under cabozantinib treatment. (Figure 4D; Figure S5B, Supporting Information). IF staining further confirmed profound marker suppression, including vasculature marker CD31 and EMCN, ECM remodeling marker fibronectin, and KDR expression. (Figure 4E).

These findings establish transformative insights that KDR governs PMN establishment and demonstrate that proactive niche disruption with VEGFR inhibitor emerges as a superior therapeutic paradigm to conventional metastasis treatment.

### 2.5 The VEGFA‐KDR Signaling Axis Orchestrates PMN Formation

While VEGFA is canonically associated with angiogenesis (promoting endothelial proliferation, migration,^[^ ^28^ ^]^ and vascular permeability^[^ ^29^ ^]^), its specific role in orchestrating PMN formation remained undefined. Building upon our discovery of Basal‐to‐CAP cell communication via VEGFA‐KDR, we implemented an integrated experimental strategy to dissect the hierarchical regulation of this pathway in PMN formation.

**Figure 5:**
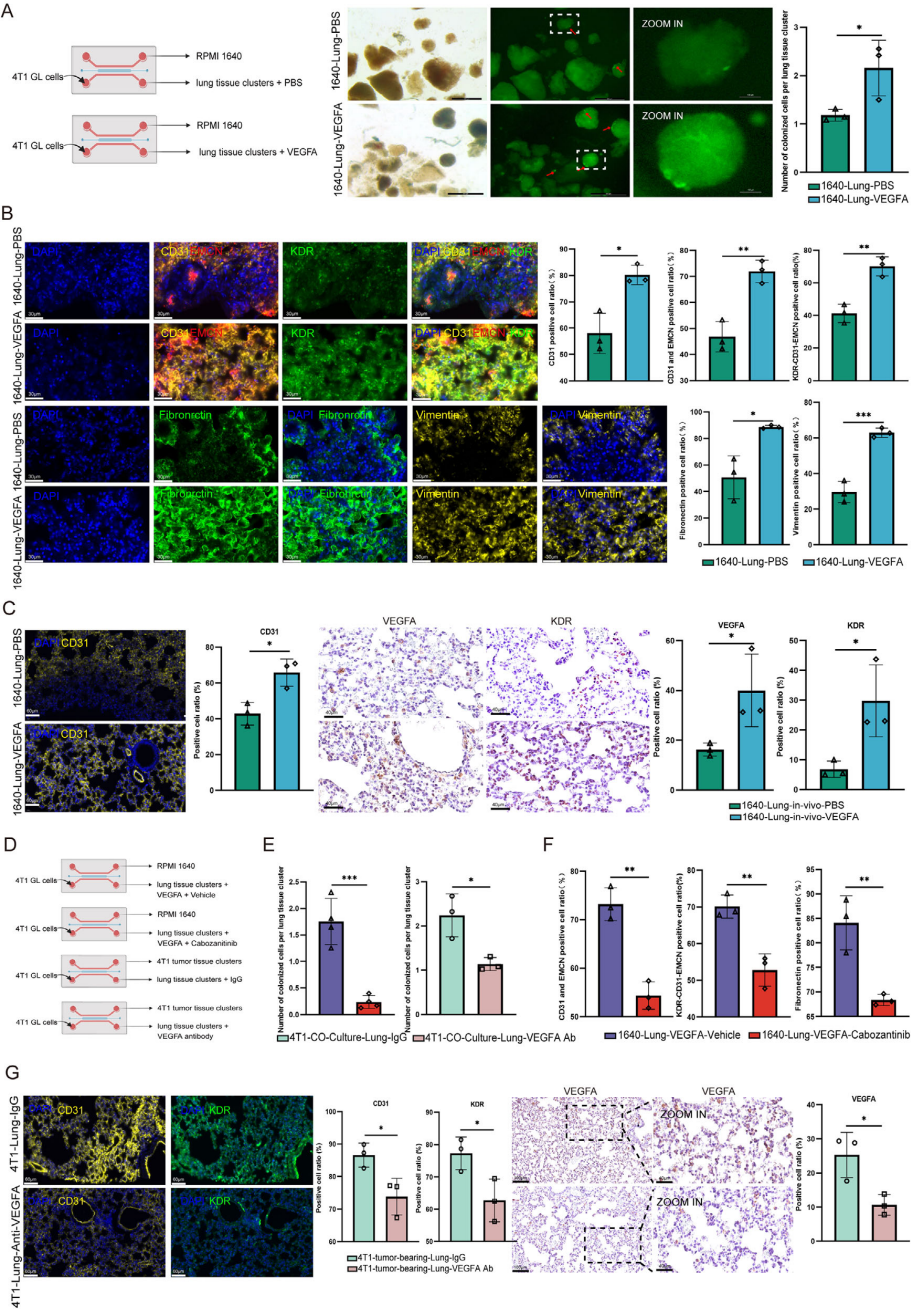
The VEGFA‐KDR Signaling Axis Drives Pulmonary PMN Formation. A) **Left**: Schematic of the micro‐organ chip‐based experimental workflow for validating VEGFA‐induced PMN formation**. Right**: Representative fluorescence images of 4T1‐GFP+ cell colonization in PBS‐ versus VEGFA‐treated lung tissue clusters and normalized colonization density (cells per cluster). (Scale bars: 500 µm; n=3; *p < 0.05, **p < 0.01, ***p < 0.001 by two‐tailed t‐test). B) Immunofluorescence analysis of lung tissues after PBS/VEGFA treatment: Nuclei (DAPI, blue), Angiogenesis (CD31, yellow), Microvasculature (EMCN, red), KDR expression (green), ECM remodeling (Fibronectin, green; Vimentin, yellow). (Scale bars: 30 µm), and quantification of CD31+, CD31+EMCN+ (CAP cells), KDR+ fractions in CAP cells, Fibronectin and Vimentin deposition. (n=3; *p < 0.05, **p < 0.01, ***p < 0.001 by two‐tailed t‐test). C) IF and IHC of in vivo lung sections from VEGFA‐infused mice showing CD31, KDR, and VEGFA expression, and marker‐positive area quantification. (Scale bars: 60 µm (IF), 40 µm (IHC); n=3 mice/group; *p < 0.05, **p < 0.01, ***p < 0.01 by t‐test). D) Schematic of the micro‐organ chip based experimental workflow for validating receptor‐dependent KDR activation, and signaling specificity of the VEGFA‐KDR axis. **Top**: 4T1‐GFP+ colonization in VEGFA protein and vehicle versus VEGFA protein and cabozantinib groups. **Bottom**: 4T1‐GFP+ colonization in tumor co‐cultured lung tissue clusters with isotype control versus αVEGFA antibody groups. E) **Left**: Normalized colonization density (cells per cluster) of 4T1‐GFP+ cell colonization in VEGFA protein and vehicle versus VEGFA protein and cabozantinib groups. **Right**: Normalized colonization density (cells per cluster) of 4T1‐GFP+ cell colonization in tumor‐lung co‐cultures with isotype control versus anti‐VEGFA. (Scale bars: 500 µm; n=3; *p < 0.05, **p < 0.01, ***p < 0.001 by two‐tailed t‐test). F) Quantification of CD31+EMCN+ (CAP cells), KDR+ fractions in CAP cells, Fibronectin deposition in VGEFA protein combine with cabozantinib or vehicle. (n=3; *p < 0.05, **p < 0.01, ***p < 0.001 by two‐tailed t‐test). G) IF and IHC analysis of lung tissues from orthotopic tumor‐bearing and VEGFA antibody or isotype control‐treated mice. Representative images of CD31 (angiogenesis), KDR (target engagement), and VEGFA (pro‐angiogenic signaling) in lung sections and quantification of marker‐positive areas. (Scale bars: 60 µm (IF), 100 and 40 µm (IHC); n = 3 mice/group; *p < 0.05, **p < 0.01, ***p < 0.001 by two‐tailed t‐test).

To validate the scRNA‐seq data, which indicated a specific upregulation of VEGFA in distantly co‐cultured pulmonary basal cells rather than in tumor cells, we performed cytokine array analyses on both supernatant from primary tumor tissues (4T1, EMT6, JC) and supernatant from lung tissues after tumor co‐culture. The cytokine array (Figure S6B, Supporting Information) revealed that VEGF secretion decreased in isolated tumor tissue after excision, but significantly increased specifically under tumor co‐cultured lung supernatant. These results support that VEGFA secretion is enhanced by lung tissue in response to tumor co‐culture, rather than by tumor tissue alone. Gain‐of‐function validation revealed that exogenous administration with recombinant VEGFA alone was sufficient to induce PMN formation, resulting in around twofold increase in tumor cell colonization (**Figure** **5**A). This was accompanied by coordinated upregulation of vascular markers (Figure 5B; Figure S6A, Supporting Information), as well as the increased expression of KDR in CAP cells. ECM components also demonstrated dramatically increasement of fibronectin and vimentin (Figure 5B).

In vivo administration of VEGFA also consistently induced dramatic pulmonary changes with CD31+ vasculature increasing and surged KDR expression (Figure 5C). However, combine treatment with a KDR inhibitor in recombinant VEGFA administration condition could abrogated the VEGFA‐mediated colonization (Figure 5D,E; Figure S6C, Supporting Information) and abolished the key PMN features, including reductions in microvascular, KDR+ CAP cells, and ECM remodeling (Figure 5F; Figure S6D, Supporting Information). Furthermore, αVEGFA antibody treatment, also attenuating tumor cell colonization in the lung in both VEGFA stimulated lung and tumor‐educated lung (Figure 5E,F; Figure S6C, Supporting Information. Concurrently, the KDR+ CAP cells and EMT markers also get abolished both in vitro and in vivo (Figure 5G; Figure S6E,F, Supporting Information).

These data crystallize VEGFA serves as the non‐redundant initiator for KDR activation, which in turn amplifies vascular niche formation and ECM remodeling – establishing the VEGFA‐KDR axis as the irreducible core of PMN programming.

### 2.6 Tumor Cells Secreted G‐CSF Promote Lung PMN Formation

**Figure 6:**
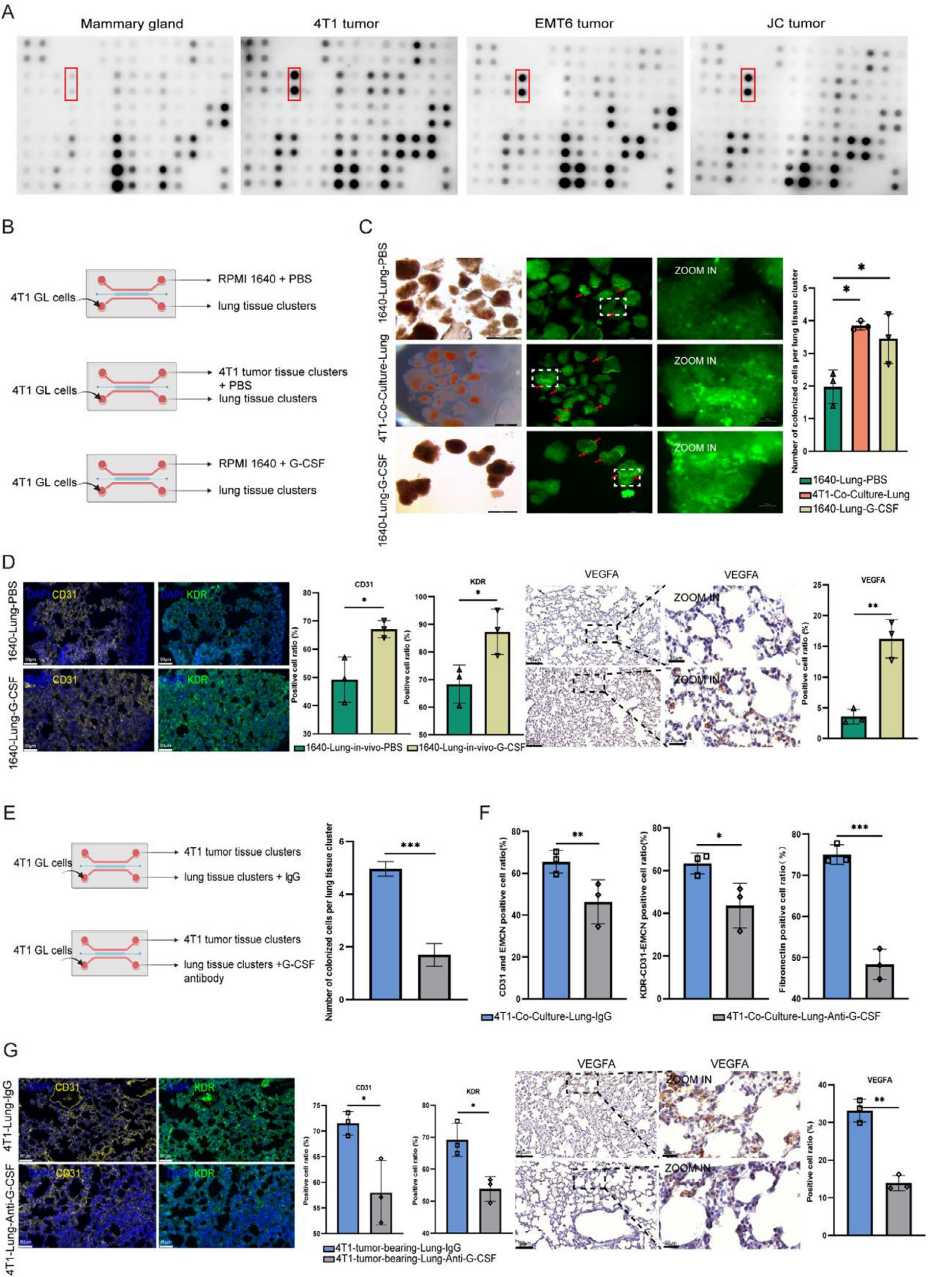
Tumor‐derived G‐CSF promotes the formation of pulmonary PMN. A) Cytokine/chemokine array profiling of normal mammary tissue versus three breast cancer subtypes (4T1, EMT6, JC), red boxes indicate the positions of G‐CSF, with each antibody array containing duplicate spots for reproducibility. B) Schematic of the micro‐organ chip experimental design for functional validation of G‐CSF. Lung tissue clusters were co‐cultured with PBS (negative control), 4T1 tumor tissue clusters (positive control), or recombinant G‐CSF. C)** Left**: Representative fluorescence images showing 4T1‐GFP+ cell colonization in lung tissues treated with PBS, 4T1 tumor clusters, or G‐CSF. **Right**: Quantification of normalized colonization density (cells per cluster). (Scale bar: 500 µm; n=3; one‐way ANOVA, *p < 0.05, **p < 0.01, ***p < 0.001). D) IF and IHC of lung sections from G‐CSF‐infused mice showing CD31, KDR, and VEGFA expression. Right: Marker‐positive area quantification. (Scale bars: 60 µm (IF), 100 and 20 µm (IHC); n=3 mice/group; *p < 0.05, **p < 0.01, ***p < 0.01 by two‐tailed t‐test). E) **Left**: Schematic of tumor co‐cultured lung tissue clusters were administrated with isotype control or αG‐CSF antibody. **Right**: Quantification of normalized colonization density (cells per cluster) about 4T1‐GFP+ cell colonization in 4T1 co‐cultured lung tissues treated with G‐CSF antibody or isotype control. (n=3; two‐tailed t‐test, *p < 0.05, **p < 0.01, ***p < 0.001). F) Quantitative assessment of CD31+EMCN+, CD31+EMCN+ KDR+, Fibronectin+ area fractions of 4T1‐preconditioned lung tissues treated with αG‐CSF antibody or isotype control. (mean ± SEM; *p < 0.05, **p < 0.01, ***p < 0.001 by two‐tailed t‐test). G) IF and IHC analysis of lung tissues from orthotopic tumor‐bearing and G‐CSF antibody or isotype control treated mice. Representative images of CD31 (angiogenesis), KDR (target engagement), and VEGFA (pro‐angiogenic signaling) in lung sections and quantification of marker‐positive areas. (Scale bars: 60 µm (IF), 100 and 20 µm (IHC); n = 3 mice/group; *p < 0.05, **p < 0.01, ***p <0.001 by two‐tailed t‐test).

To identify key tumor‐secreted mediators of PMN development, we performed cytokine profiling comparing three breast cancer models (4T1, EMT6, JC) to normal mammary tissue. Cytokine array analysis revealed granulocyte colony‐stimulating factor (G‐CSF/CSF3) as the most consistently upregulated factor across all tumors (**Figure** **6**A **;** Figure S7A, Supporting Information).

Functional validation in the micro‐organ chip system (Figure 6B) demonstrated that recombinant G‐CSF alone recapitulated of tumor coculture‐induced PMN remodeling effects on tumor cell colonization (Figure 6C). Notably, the effects of G‐CSF treatment showed no statistically significant difference from those observed in 4T1 coculture condition, establishing G‐CSF as the dominant tumor‐derived mediator of PMN induction. This functional dominance was further confirmed by αG‐CSF antibody neutralization, which significantly reduced colonization compared to IgG controls (Figure 6E; Figure S7D, Supporting Information).

IF analysis shows G‐CSF stimulates the master molecular KDR in metastatic niche programming. As predicted, PMN characterization revealed expansion of CD31+ vasculature and an increase in CD31+EMCN+ microvessels (Figure S7B, Supporting Information), as well as orchestrates ECM reorganization, with upregulation of fibronectin vimentin (Figure S7C, Supporting Information). Conversely, G‐CSF neutralization significantly attenuated the PMN features, resulting in reduction in CD31+EMCN+ microvessels (from 65.52% to 46.38%), decrease in KDR+ CAP cells (from 63.33% to 43.63%), and suppression of ECM markers (Fibronectin: from 75.06% to 48.36%) (Figure 6F; Figure S7E, Supporting Information).

Physiological conservation was observed in mouse model validation. G‐CSF administration, inducing angiogenesis increase, KDR upregulation, and dramatic VEGFA elevation (around twofold) in pulmonary tissue (Figure 6D). Mirroring this, αG‐CSF therapy in tumor‐bearing mice demonstrates a decreased angiogenesis deposition (CD31⁺ vessels from 71.54% to 57.99%), KDR expression (from 69.17% to 53.86% and VEGFA (from 33.20% to 13.91%) (Figure 6G).

Collectively, these data establish G‐CSF drives pulmonary pre‐metastatic niche formation to facility the tumor cell colonization by regulating pulmonary vascular/microvascular generation and promoting ECM remodeling.

### 2.7 Tumor‐Secreted G‐CSF Orchestrates Pulmonary PMN Formation via the VEGFA‐KDR Axis

**Figure 7:**
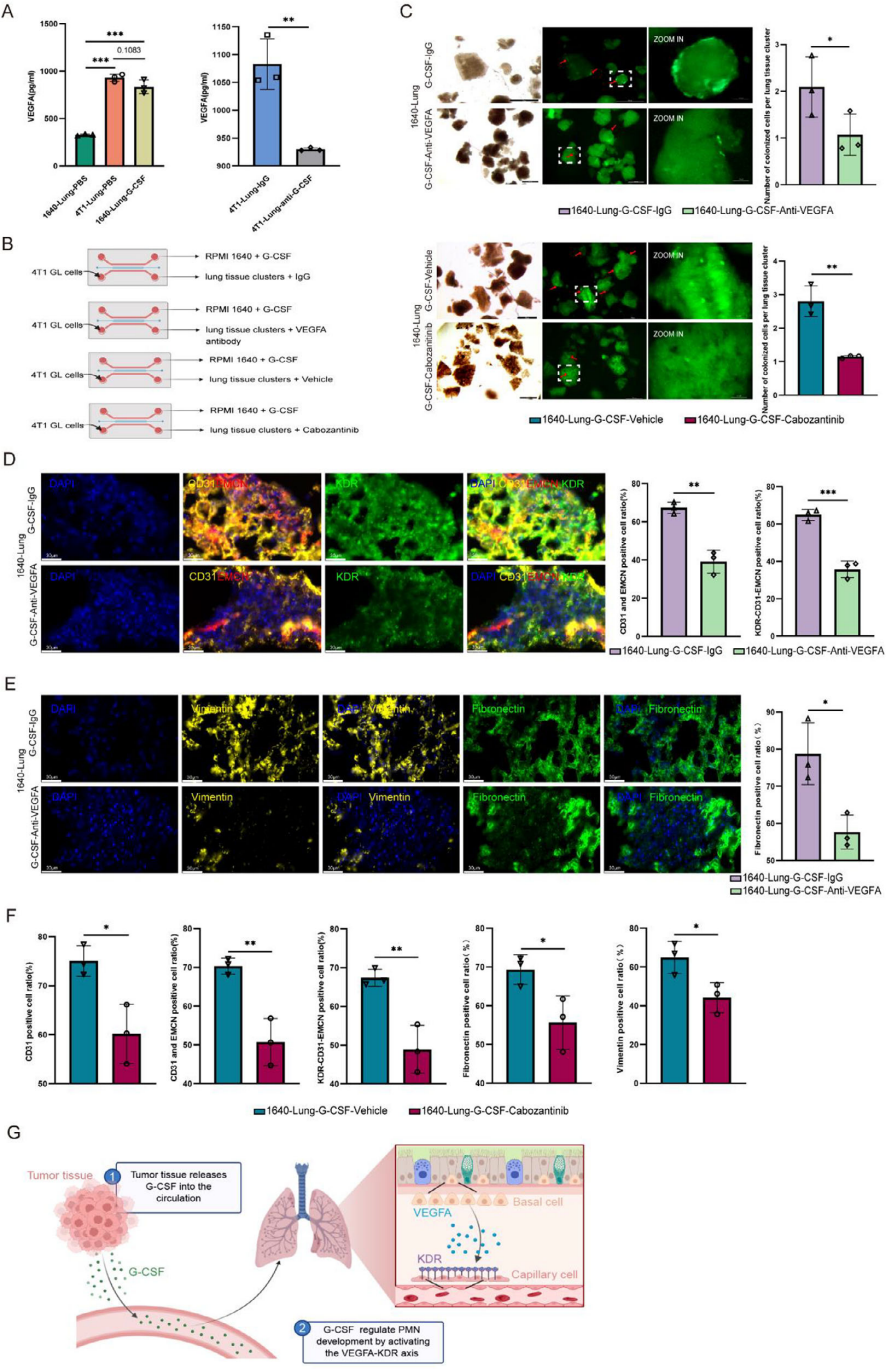
Tumor‐derived G‐CSF regulates VEGFA‐KDR axis. A) ELISA quantification of VEGFA levels in conditioned media from differentially treated lung tissues. Tumor co‐culture and recombinant G‐CSF significantly increased VEGFA secretion compared to RPMI 1640 controls (n=3; *p < 0.05, **p < 0.01, ***p < 0.001 by one‐way ANOVA).B) Schematic of the experimental design for functional validation of G‐CSF‐mediated VEGFA‐KDR axis regulation in the micro‐organ chip system. Key interventions included: VEGFA neutralization (VEGFA antibody) and KDR inhibition (cabozantinib). C) Representative fluorescence images and quantitative analysis of 4T1‐GFP+ cell colonization in lung tissues treated with G‐CSF plus VEGFA antibody and parallel experiments with G‐CSF plus cabozantinib. Normalized colonization density (cells per cluster) demonstrates a significant reduction in tumor cell adhesion upon pathway disruption (Scale bar: 500 µm; n=3; two‐tailed t‐test, *p < 0.05, **p < 0.01, ***p < 0.01). D) Immunofluorescence analysis of lung tissues post‐treatment with G‐CSF combine with VEGFA antibody. Nuclei (DAPI, blue), angiogenesis (CD31, yellow), microvasculature (EMCN, red), and KDR expression (green). (Scale bars: 30 µm), and quantification of CD31+EMCN+ (CAP cells), KDR+ fractions in CAP cells. E) Immunofluorescence analysis of lung tissues post‐treatment with G‐CSF combined with VEGFA antibody. Nuclei (DAPI, blue), ECM remodeling (Fibronectin, green; Vimentin, yellow). (Scale bars: 30 µm), and quantification of Fibronectin deposition. F) Quantitative analysis of CD31+, CD31+EMCN+ (microvascular/CAP cells), KDR+ CAP cells, Fibronectin, and Vimentin deposition in G‐CSF‐cabozantinib. (n=3; two‐tailed t‐test, *p < 0.05, **p < 0.01, ***p < 0.001). G) Schematic illustration of how tumor‐secreted G‐CSF promotes pre‐metastatic niche formation in the lung by modulating the VEGFA‐KDR signaling axis.

Then we validate whether G‐CSF regulates PMN programming through VEGFA‐KDR axis. ELISA analysis of conditioned media revealed G‐CSF alone induced a striking increase of VEGFA production in lung tissue channel, which is consistent with 4T1 tumor coculture (**Figure** **7**A). Crucially, this G‐CSF‐dependent VEGFA induction was significantly attenuated by αG‐CSF neutralizing antibody treatment establishing G‐CSF as a critical upstream regulator of VEGFA production.

To further validate this hierarchical signaling network in PMN architecture, we conducted perturbation experiments in our micro‐organ chip platform (Figure 7B). VEGFA antibody neutralization produced profound effects across multiple PMN parameters, including reduction in tumor cell colonization (Figure 7C), accompanied by significant decreases in vascularization, CAP cell abundance, and KDR expression (Figure 7D; Figure S8A, Supporting Information). Furthermore, this intervention markedly attenuated ECM remodeling processes fibronectin decreased from 78.79% to 58.69% and vimentin deposition from 65.98% to 43.31%, respectively (Figure 7E; Figure S8A, Supporting Information). Parallel experiments with the KDR inhibitor cabozantinib yielded complementary results, which demonstrating suppression of tumor cell adhesion (Figure 7C) along with coordinated decreases in vascular (CD31+: from 75.05% to 60.19%; CD31+EMCN+: from 70.32% to 50.75%), and ECM structural (vimentin+: from 64.98% to 44.28%) markers (Figure 7F; Figure S8B,C, Supporting Information). The striking concordance between VEGFA blockade and KDR inhibition outcomes provides compelling evidence for a G‐CSF→VEGFA→KDR signaling cascade governing PMN establishment.

In summary, our integrated analyses establish that tumor‐secreted G‐CSF drives breast cancer lung metastasis by activation of the VEGFA‐KDR axis, which orchestrates three fundamental PMN maturation processes: 1) angiogenic specialization through microvascular expansion, 2) structural remodeling via fibronectin and vimentin deposition, and 3) functional reprogramming of CAP cells. The mechanistic dissection of this pathway hierarchy, achieved through systematic combinatorial perturbations, not only advances our understanding of PMN biology but also identifies the G‐CSF‐VEGFA‐KDR axis as a promising therapeutic target for metastasis prevention.

## 3 Discussion

Our study establishes a groundbreaking 3D tumor–lung coculture model using a micro‐organ chip platform, which effectively simulates the remote interactions between tumor and lung tissues and enables the recapitulation of dynamic pre‐metastatic niche (PMN) formation in breast cancer lung metastasis ex vivo. As reviewed by Zhang et al., 3D models demonstrate unique advantages in dissecting intercellular communication and the mechanisms of tumor metastasis.^[^ ^30^ ^]^ By leveraging the temperature‐dependent phase transition properties of type I collagen, we engineered a microfluidic barrier system that simultaneously maintains 3D tissue growth while achieving fluidic isolation. This design allowed us to precisely dissect tumor paracrine signaling in PMN induction while eliminating confounding effects from direct tumor cell migration. Three key discoveries emerge from our work: 1) Tumor cells remotely regulate PMN formation through secreted factors‐lung tissues co‐cultured with tumors demonstrated significantly enhanced tumor colonization capacity despite physical segregation; 2) G‐CSF serves as a master regulator of PMN development by activating the VEGFA‐KDR axis in lung basal cells, thereby driving pro‐angiogenic phenotypic transformation of capillary (CAP) cells; and 3) PMNs exhibit broad‐spectrum receptivity, supporting metastasis across different breast cancer subtypes–a finding with important implications for developing universal anti‐metastatic therapies.

Through single‐cell RNA sequencing, we identified upregulation of KDR in CAP cells as a defining characteristic of PMNs. While VEGFA secreted by basal cells directly modulates this process as the canonical KDR ligand, our work innovatively positions tumor‐derived G‐CSF as the initiating factor of this signaling cascade. According to previous studies, G‐CSF promotes tumor metastatic progression by mobilizing pro‐angiogenic myeloid cells.^[^ ^31^ ^]^ (2) activating the STAT3/β1‐integrin signaling pathways,^[^ ^31^ ^]^ inducing neutrophil extracellular trap (NET) formation, and enhancing the epithelial‐mesenchymal transition (EMT) processes.^[^ ^32^, ^33^, ^34^ ^]^ Our discovery extends previous knowledge about VEGFA‐KDR‐mediated angiogenesis^[^ ^35^ ^]^ by establishing G‐CSF as the central orchestrator of the “G‐CSF→VEGFA→KDR” signaling axis that confers sustained activation potential to PMNs. Importantly, whereas prior studies have emphasized the roles of immune cells^[^ ^36^ ^]^ and bone marrow components^[^ ^37^ ^]^ in PMN formation, our micro‐organ chip system demonstrates that tumor tissues alone can initiate PMN development. This paradigm‐shifting finding redefines the temporal sequence of molecular events in PMN establishment and identifies an earlier therapeutic window for intervention.

While our micro‐organ chip platform effectively demonstrates the autonomous capacity of tumor tissues to initiate PMN formation, it remains a simplified representation of the in vivo milieu. Notably, the absence of systemic immune components and the inability to model multi‐organ crosstalk represent key limitations of our current system. These constraints underscore the inherent gap between our reductionist model and the organism‐level complexity of metastatic dissemination.

To further advance our understanding of metastatic processes, future studies should focus on: 1) Developing multi‐channel chips incorporating immune and bone marrow components to better approximate the in vivo microenvironment while maintaining experimental control; and 2) Elucidating whether STAT3/NF‐κB pathways participate in G‐CSF signal transduction. The platform developed here also enables high‐throughput screening of G‐CSF/KDR dual‐target inhibitors, potentially overcoming current limitations in anti‐metastatic therapy.

We propose a novel paradigm in which tumors remotely “educate” distant lung CAP cells through G‐CSF secretion, driving PMN formation via the VEGFA‐KDR axis. Clinically, combined blockade of G‐CSF and KDR may represent a promising new strategy for preventing pulmonary metastasis. Beyond these mechanistic insights, our study provides a transformative model system, the 3D tumor‐lung coculture platform, that establishes a new approach for investigating metastasis mechanisms and screening therapeutic interventions.

## 4 Experimental Section

### Micro‐Organ Chip Design and Fabrication

The micro‐organ chip platform was designed using Autodesk Fusion 360 software to create both 2D schematics and 3D architectural models (Figure S1A, Supporting Information). High‐precision molds were fabricated using biocompatible resin through 3D printing technology. For chip fabrication, polydimethylsiloxane (PDMS; SYLGARD‐184, Dow Corning) prepolymer and cross‐linker were precisely mixed at a 20:1 (w/w) ratio, followed by vacuum degassing. The mixture was then cast onto 3D‐printed resin molds with micron‐scale resolution. Thermal curing was performed at 60 °C for 12 h to achieve optimal mechanical stability while maintaining tissue‐level compliance. After demolding, both the PDMS structure and alignment fixture (50 µm thickness) underwent oxygen plasma treatment (100 mTorr, 30 W, 45 sec) to enable irreversible bonding. The assembled device was subsequently baked at 80 °C for 15 min to enhance adhesion and ensure leak‐proof operation. For sterilization, the bonded PDMS micro‐organ chip was autoclaved at 121 °C for 20 min.

To establish a physiological barrier separating tumor and lung tissue compartments, a RAT Collagen I matrix (R&D, 2440‐100‐01) was prepared by mixing 10x F‐12 nutrient mixture (Thermo Fisher 21700075) and Collagen buffer (260 mm NaHCO_3_, 20 mm HEPES, 0.05N NaOH) in a volume ratio of 7:1:2 (pH 7.0–7.5). The resulting mixture (3.5 mg mL^−1^) was injected into the central channel and polymerized at 37 °C (5% CO_2_) for 2 h to form a solid barrier. To simulate hemodynamic conditions and enable continuous low‐flow perfusion of tumor cells, the assembled micro‐organ chips were placed on a shaker operating at 20 rpm with 3D rotation. The entire setup was maintained in a cell culture incubator to ensure optimal growth conditions for both tissues and cells throughout the experiments.

### Cell and Tissue Cluster Culture

JC (RRID: CVCL_3530, CRL‐2116, TIN HANG TECHNOLOGY LIMITED, 21 April 2020), 4T1 (RRID: CVCL_0125) and EMT6 (RRID: CVCL_1923) cell line were kindly gifted to the laboratory by Professor Chuxia Deng’ lab, University of Macau. To generate 4T1‐GL, EMT6‐GL, or JC‐GL cells, the tumor cells were infected with GFP and luciferase‐expressing lentivirus (Addgene). All cell lines were tested and verified to be free of mycoplasma contamination using a PCR‐based method prior to and during the study. All cells and tumor tissue clusters were cultured in Roswell Park Memorial Institute (RPMI) 1640 medium with 10% FBS (A5256701, Gibco, Waltham, MA, USA) and 1% penicillin‐streptomycin (Invitrogen, Carlsbad, CA).

Mice were euthanized by cervical dislocation under aseptic conditions. Orthotopic breast tumors and lungs from normal control mice were excised and immediately rinsed in ice‐cold PBS (pH 7.4, Gibco). Tissues were transferred to a biosafety cabinet and minced into fragments using sterile surgical scissors. To prevent microchannel clogging and ensure experimental reproducibility, minced tissues were sequentially filtered through 400 and 150 µm cell strainers. Tissue clusters within the 150–400 µm size range were collected for culture. Lung tissue clusters were cultured in Ham's F‐12‐Dulbecco's modified Eagle's medium (DMEM/F12) (11330, Gibco, Waltham, MA, USA), supplemented with 10% fetal bovine serum (FBS) (A4766801, Gibco, Waltham, MA, USA) and 1% penicillin–streptomycin (Invitrogen, Carlsbad, CA). Tumor tissue clusters were cultured in RPMI 1640 medium with 10% FBS and 1% penicillin‐streptomycin (Invitrogen, Carlsbad, CA).

### Animal Models

For all animal work, mice with similar age and weight were randomized before tumor inoculation. 4T1/EMT6/JC murine mammary carcinoma cells (1×10^6 cells in 50 µL PBS) were injected into the fourth mammary fat pad of 6–8‐week‐old female BALB/c mice (Charles River Laboratories). Seven days post‐implantation, GFP‐ and luciferase‐labeled tumor cells (1×10^5 cells/mouse in 100 µL PBS) were administered via tail vein injection to mimic hematogenous dissemination. Cell viability was confirmed to be >95% by Trypan Blue exclusion prior to injection. Prior to further treatment, the tumor‐bearing mice were randomized with respect to their tumor sizes to ensure all treatment groups had equivalent tumor burden before treatment. All animal experiments were performed in the same well‐controlled pathogen‐free facility with the same mouse diets.

### Tissue Clusters Viability Assay

Alamar Blue (DAL1025, Thermos Fisher Scientific, Alamar Blue Cell Viability Reagent) incubate was used with tissue cluster at 37 °C for 2 h on a shaker to ensure full mixing. The samples were centrifuged at 500 g for 3 min, and the absorptivity values of the supernatant were measured at 560 and 595 nm to characterize the lung tissue cluster viability.

### Tumor GL Cells Quantification

All lung tissue clusters were harvested and washed three times with PBS (centrifugation at 500 g for 5 min) to remove non‐adherent tumor GL cells. The lung tissue clusters were then collected and the number of GL cells was quantified using stereo fluorescence microscope (M165 FC Fluorescent Stereo Microscope). The ratio of all GL cells adhering to lung tissue observed under the microscope to the total collected lung tissue was used as the basis for subsequent analysis.

### Single Cell Sequencing

Transfer all lung tissue from the lung channel to 1.5 mL tube using DMEM/F12 complete medium. Wash the tissue three times with pre‐cooled PBS by centrifugation (500 g × 5 min) and discard the supernatant. Add 1 mL of tissue lysis buffer and incubate in a shaking incubator at 37 °C (300 rpm, 30–60 min). The lysis buffer was prepared by adding 0.1 g of collagenase type II (17101015, Thermos Fisher Scientific, Waltham, MA, USA) and 0.1 g of collagenase type IV (17104019, Thermos Fisher Scientific, Waltham, MA, USA) to 50 mL of PBS. Mix by pipetting with a 1 mL pipette every 20 min until no tissue fragments remain. Transfer the suspension to a 50 mL centrifuge tube and neutralize the lysis buffer with 15 mL of DMEM/F12 medium. Filter the suspension through a 70 µm cell strainer, centrifuge the filtrate (2500 rpm, 5 min), and discard the supernatant. Add 3‐5 times the volume of red blood cell lysis buffer (C3702, Beyotime Biotechnology, Shanghai, China) and lyse at room temperature for 1–2 min. Terminate the lysis by adding 2 times the volume of culture medium, centrifuge at 500 g for 5 min, and discard the supernatant. Wash the pellet three times with PBS, stain the cells with trypan blue, and count them using a cell counter. Single‐cell RNA libraries were prepared using the 10x Chromium Single Cell platform using the Chromium Single Cell 3′ Library, Gel Bead and Multiplex Kit, and Chip Kit (10x Genomics, Pleasanton, CA, USA).

### Elucidating Key Signaling Pathways Regulating PMN Formation Through Integrated In Vitro and In Vivo Models

To model tumor–lung interactions, a micro‐organ chip platform was established that integrated mouse lung tissue with either co‐cultured tumor tissue or blank RPMI 1640 medium as a control. For pharmacological interventions, the system was treated with cabozantinib malate (XL184, 1.5 µm; Selleck) or vehicle (DMSO), recombinant VEGF164 (5 ng µL^−1^; MedChemExpress), anti‐mouse VEGF‐A neutralizing antibody (2G11‐2A05, 1 ng µL^−1^; Bio X Cell), recombinant G‐CSF (10 ng µL^−1^; Servicebio), or anti‐mouse G‐CSF antibody (MAB414, 1 ng µL^−1^; R&D Systems). Parallel in vivo validation studies were performed in mouse xenograft models using intraperitoneal administration of cabozantinib malate (20 mg kg^−1^ twice weekly), recombinant VEGF164 (0.5 mg kg^−1^), anti‐mouse VEGF‐A (5 mg kg^−1^), recombinant G‐CSF (2.5 mg kg^−1^), or anti‐mouse G‐CSF (1 mg kg^−1^), with doses selected based on established protocols to achieve target inhibition while maintaining tolerability.

### Histological Analyses and Immunohistochemistry

Lung tissues were fixed in 10% neutral buffered formalin (NBF) for 24 h to preserve morphology and antigen integrity. After fixation, tissues were dehydrated in a graded ethanol series and embedded in paraffin using a Tissue‐Tek VIP processor (Sakura Finetek). Tissue microarrays (TMAs) were prepared in a 20‐well format and incubated at 40 °C overnight for optimal paraffin fusion. Sections (5 µm) were cut using a Leica RM2255 microtome and mounted on Superfrost Plus slides (Thermo Fisher Scientific), followed by drying at 48 °C overnight.

Sections were deparaffinized in xylene and rehydrated in a descending ethanol series. After PBS washes, antigen retrieval was performed in citrate buffer (pH 6.0) at 95 °C for 20 min using a decloaking chamber (Biocare Medical). Endogenous peroxidase activity was blocked with 3% H_2_O_2_ in methanol (20 min, RT), followed by incubation with 5% BSA (Sigma–Aldrich) for 30 min to reduce nonspecific binding. Primary antibodies—anti‐VEGFR2 (ab315238, Abcam; 1:200), anti‐VEGFA (HY‐P80929, MedChemExpress; 1:150), and anti‐CD31 (ab182981, Abcam; 1:100)—were applied overnight at 4 °C. HRP‐conjugated secondary antibodies (Vector Laboratories) were incubated for 1 h at RT, and signals were amplified using the VECTASTAIN Elite ABC‐HRP Kit (Vector Labs). Diaminobenzidine (DAB; Dako) was used for detection (5 min), followed by counterstaining with Mayer's hematoxylin (1 min). Slides were dehydrated, cleared in xylene, and mounted with Permount (Fisher Scientific).

### Immunofluorescence (IF) Staining of Lung Tissue Microarrays (TMAs)

Formalin‐fixed, paraffin‐embedded (FFPE) lung tissues from 60 cores (1 mm diameter per core) were arrayed into a recipient paraffin block using a tissue microarrayer. 5‐µm‐thick sections were mounted on charged slides and baked at 60 °C for 1 h. Paraffin sections were deparaffined, rehydrated, blocked with 5% goat serum and then incubated overnight at 4 °C with primary antibody against CD31 (ab182981, Abcam; 1:100), EMCN (sc‐65495, Santa Cruz; 1:50), Fibronectin (ab2413, Abcam; 1:100), Vimentin (ab92547, Abcam; 1:100), KDR (sc‐6251, Santa Cruz 1:50; dilution). After blocking with 5% bovine serum albumin (BSA; Sigma–Aldrich) for 1 h at room temperature (RT), sections were incubated with primary antibodies overnight at 4 °C. Species‐matched secondary antibodies conjugated to Alexa Fluor dyes (Invitrogen) were applied for 1 h at RT in the dark: Alexa Fluor 594 donkey anti‐rabbit (1:500), Alexa Fluor 647 donkey anti‐mouse (1:500), Alexa Fluor 488 donkey anti‐rabbit (1:500), Alexa Fluor 555 donkey anti‐rabbit (1:500), and Alexa Fluor 488 donkey anti‐mouse (1:500). Nuclei were counterstained with Hoechst 33342 (Invitrogen, 1:1000). Photomicrographs were taken with a confocal laser‐scanning microscope, using QImaging digital camera and Imaging ProPlus software. All images were captured using similar confocal microscopy settings.

### ELISA

In the micro‐organ chip system, conditioned media from lung tissues exposed to various treatments were collected and centrifuged at 500 × g for 5 min to remove tissue cluster debris. The resulting supernatants were then analyzed by enzyme‐linked immunosorbent assay (ELISA) to quantify target protein concentrations. ELISA experiments (VEGFA, SEA143Mu from CLOUD CLONE) was carried out according to the manufacturer's instructions.

### In Vivo Mouse Imaging

A 15 mg mL^−1^ D‐luciferin stock solution was prepared in sterile DPBS and sterilized by filtration through a 0.2 µm filter. Lung metastasis was tracked twice weekly by bioluminescence imaging (IS1821N7428, PerkinElmer, USA) after intraperitoneal injection of D‐luciferin (150 mg kg^−1^). Mice were sacrificed at defined endpoints (3‐15 days post‐injection) for ex vivo analysis.

### Cytokine and Chemokine Array Test

To systematically characterize the secretory profile of tumor and mammary tissues, comprehensive cytokine screening was performed using a commercial antibody array kit (Mouse Cytokine Array C1000, AAM‐CYT‐1000‐8, RAY Biotech) according to the manufacturer's protocol. Freshly isolated tumor tissue clusters and normal mammary gland tissues were minced into 1 mm^3^ fragments in ice‐cold PBS. Tissue fragments were centrifuged (500x g, 10 min, 4 °C) and supernatants were diluted before cytokine array determination. Membranes were blocked with blocking buffer for 30 min at room temperature. 1 mL of diluted sample was incubated with membranes overnight at 4 °C with gentle agitation. After washing, biotin‐conjugated detection antibodies were applied for overnight at 4 °C. Streptavidin‐HRP (1:2000) was added for 2 h at room temperature. Chemiluminescent signals were captured using a CCD imaging system (ChemiDoc MP, Bio‐Rad) and quantified with ImageLab 6.0 software. Using positive controls and negative controls to have inter‐array normalization and background subtraction.

### Ethics Approval Statement

All mouse experiments were performed under the ethical guidelines of the University of Macau (animal protocol number: UMAEC‐015‐2019, UMARE‐023‐2022).

## Conflict of Interest

The authors declare no conflict of interest.

## Author Contributions

J.Z., X.H., and L.M. contributed equally to this work. K.M. designed this study; K.M., J.Z. and L.W. established the tumor lung co‐culture system (micro‐organ chip); X.H. and Y.G. performed bioinformatics analyses; K.M., J.Z., L.M., L.W., Z.C., T.L., and H.X. performed experiments; J.Z., L.M., and Z. C. conducted the cell colonization experiments and data analysis; J.Z. and X.H. performed cell‐cell communication analysis and the bioinformatics analysis; J.Z., L.M., L. W., and Z.C. collected in vivo and in vitro samples for immunostaining and analysis. K.M., Y. C., J. L. J. Q., and X. W. supervised the experiments; and K.M. and J. Z. wrote the manuscript.

## Supporting information



Supporting Information

## Data Availability

The data that support the findings of this study are available from the corresponding author upon reasonable request.
