# Supplementary material for: Micro‐Organ Chip Deciphers Tumor‐Derived G‐CSF as Remote Commander of Lung Pre‐Metastatic Niche via VEGFA‐KDR Cascade
Source: Adv Sci (Weinh). 2025 Nov 22;13(7):e18584. doi: 10.1002/advs.202518584 (PMC12866876; doi:10.1002/advs.202518584)
Supplement: Supplementary file 1 — Supporting Information [file ADVS-13-e18584-s001.docx]

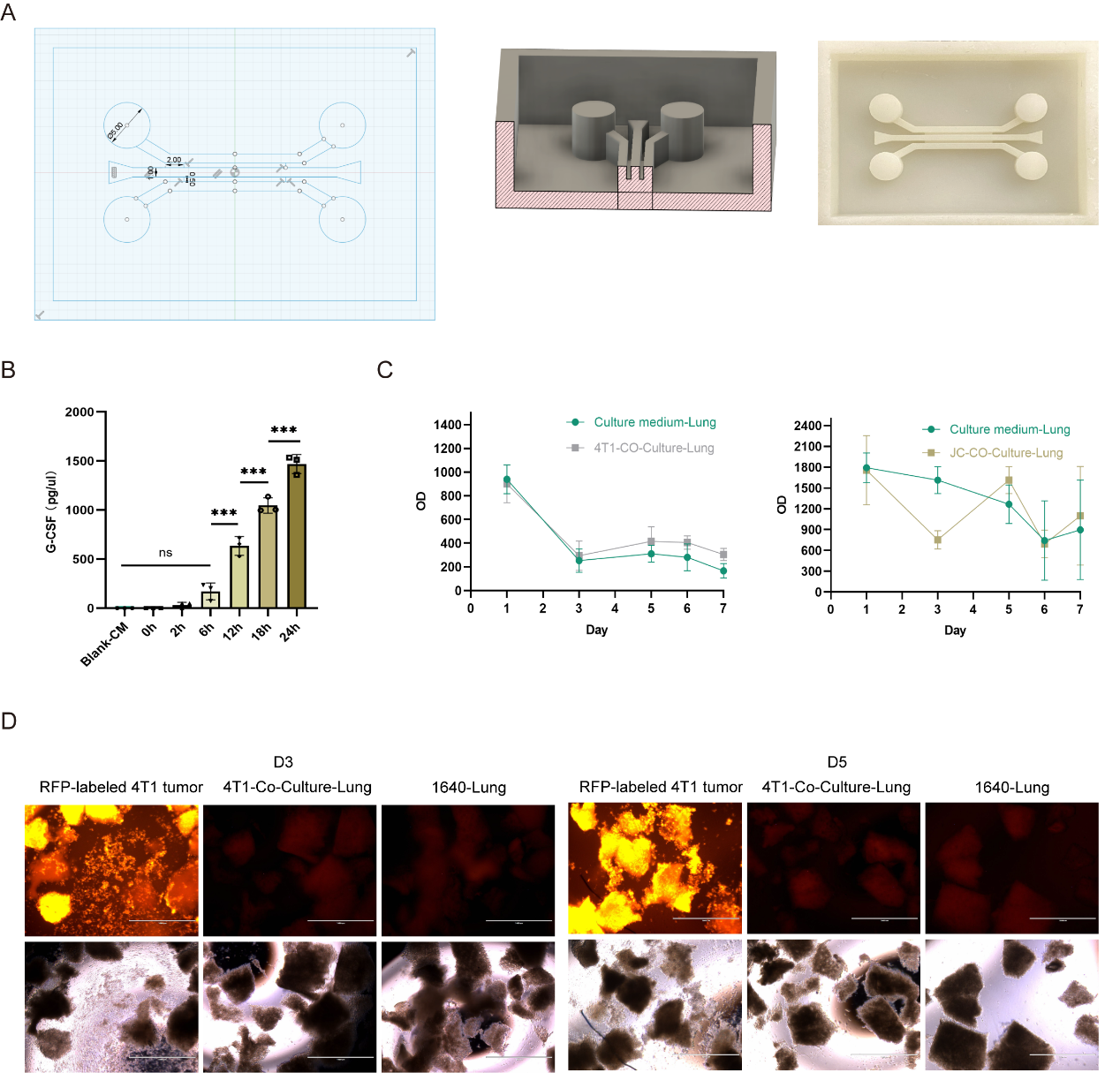


**Figure S1. Micro-organ chip design and functional validation.** **(A)** Computational design of the micro-organ chip architecture **(Left and Middle)**, additive manufacturing of the micro-organ chip mold via 3D printing **(Right)**. **(B)** Quantification of G-CSF protein concentration in the lower channel by ELISA. Blank-CM indicates the control group with blank RPMI-1640 complete medium. Data points from 0 to 24 h represent G-CSF levels measured in the lower channel at different time points after the addition of G-CSF to the upper channel. (n=3 biological replicates; NS: not significant; **P*<0.05, ***P*<0.01, ****P*<0.001 by one-way ANOVA).  **(C)** The OD value of different treated lung tissue clusters and tumor tissue clusters from one days to seven days (n=3). **(D)** Time-resolved fluorescence imaging of 4T1-RFP diffusion between adjacent tissue chambers (Day 3 to day 5 post-barrier maturation, Scale bars, 1000 µm).


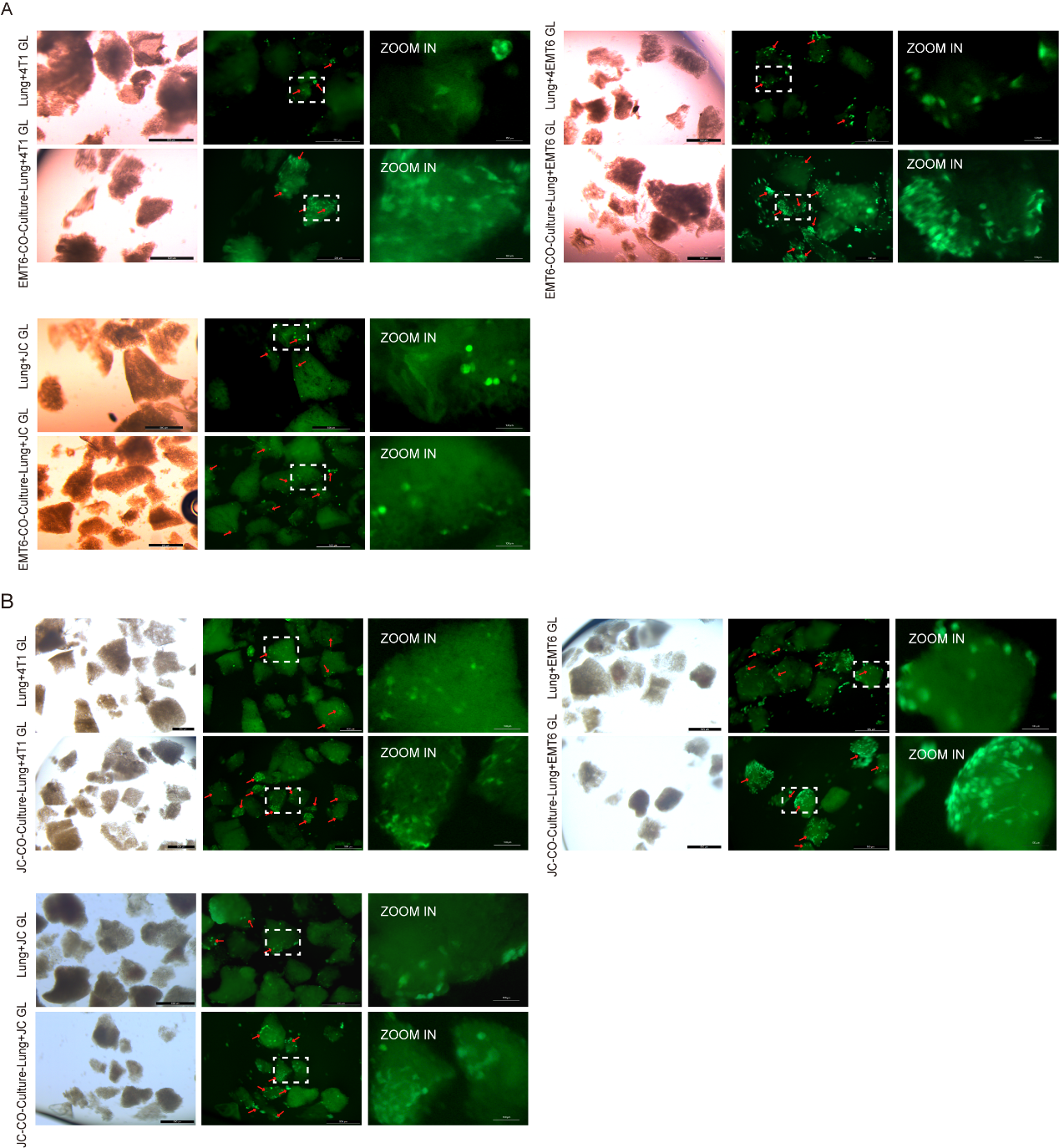


**Figure S2. Validation of tumor pulmonary PMN formation****. (A)** Representative fluorescence images of 4T1-GL, EMT6-GL, and JC-GL cell colonization in lung tissues co-cultured with EMT6 tumor tissues. (Scale bar: 500 μm). **(B)** Representative fluorescence images of 4T1-GL, EMT6-GL, and JC-GL cell colonization in lung tissues co-cultured with JC tumor tissues. (Scale bar: 500 μm).


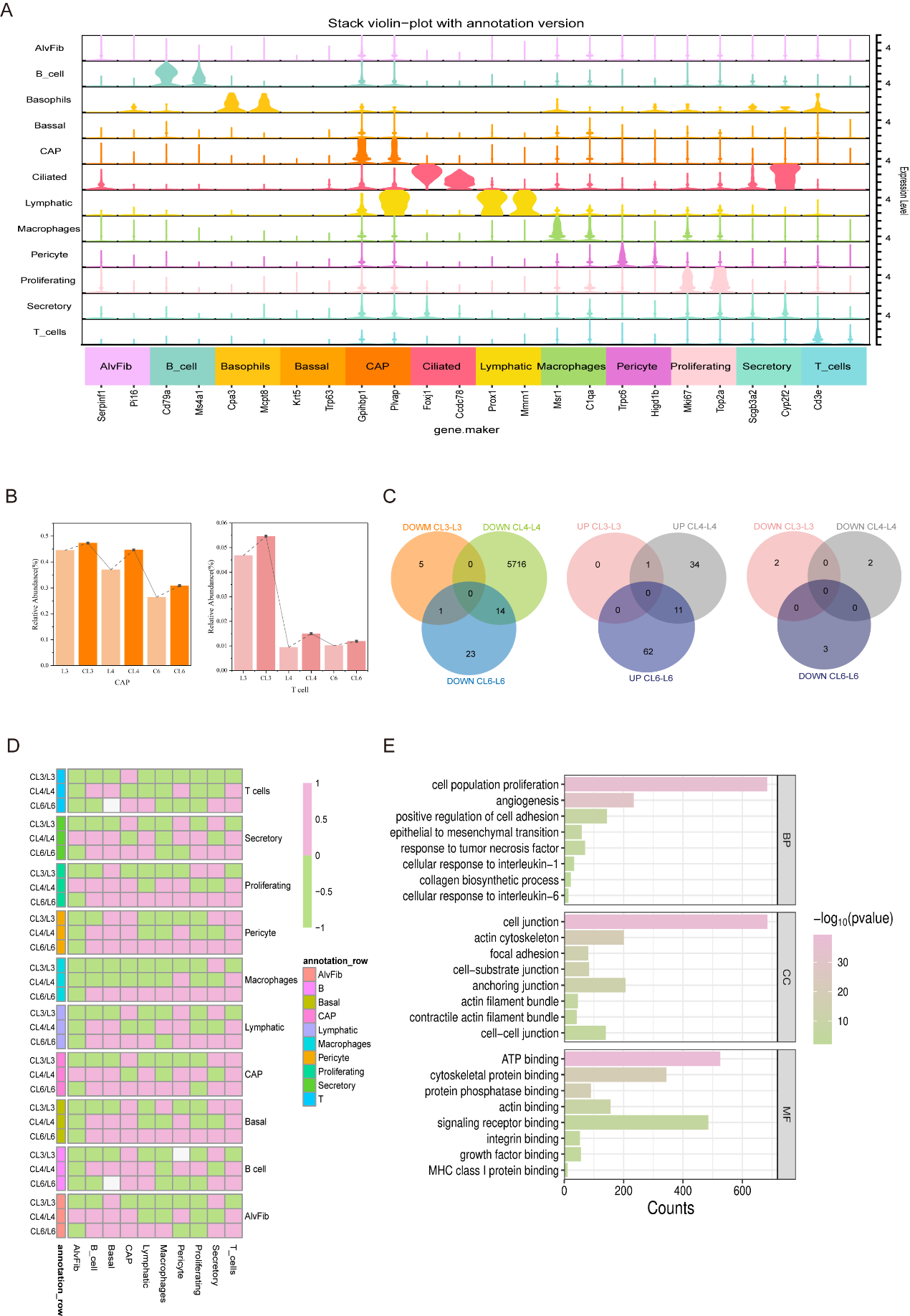


**Figure S3. Single-cell RNA sequencing identifies candidate gene in PMN formation.** **(A)** Violin plots showing marker gene expression profiles used for classifying 12 distinct cell populations in lung tissues. **(B)** Dynamic changes in cellular composition in tumor-co-cultured lung tissues (CLN) vs. controls (LN) across time points. Quantification of CAP cells **(Left)** and T cells **(Right)**, these two populations showing consistent expansion trends. **(C)** Three Venn diagrams compare overlapping differentially expressed genes (DEGs) in CAP and T cells. **Left**: CAP cell downregulated DEGs shared among CL3/L3, CL4/L4 and CL6/L6 conditions; **Middle**: T cell upregulated DEGs common to all conditions; **Right**: T cell downregulated DEGs present across all three condition pairs. **(D)** Heatmap of cell-cell interaction strength. CLN/LN ratios depict increased (pink) or decreased (green) interactions in co-cultured vs. control lungs (Y-axis: ligand-expressing cells; X-axis: receptor-expressing cells). **(E)** Gene Ontology (GO) enrichment analysis of 82 up-regulated DEGs in CAP cells.


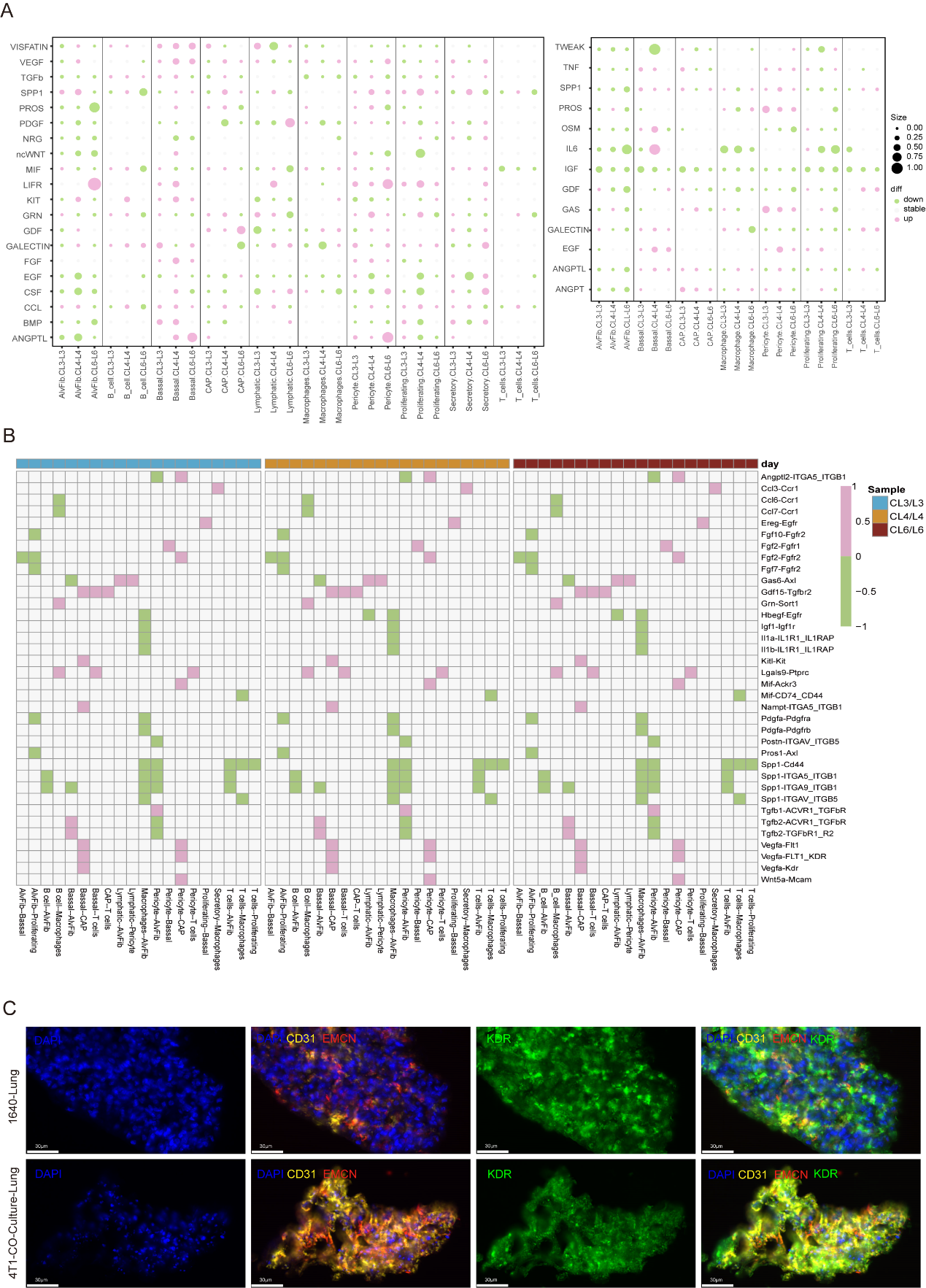


**Figure S4. Single-cell RNA sequencing identifies candidate gene in PMN formation.** **(A)** Bubble plot depicting cytokine signals received by target cells (**Left**) and secreted cytokine profile of source cells (**Right**). **(B)** Heatmap of differentially expressed ligand–receptor interaction pairs among cell subtypes at different time points during PMN formation. The Y-axis displays specific ligand-receptor pairs (annotated by gene symbols), while the X-axis represents interacting cell-type pairs. The color scale indicates relative interaction strength (based on average expression of ligands and receptors), with pink denoting upregulated interactions and green representing downregulated interactions. **(C)** Immunofluorescence staining of lung tissue clusters showing of KDR expression (green) in CAP cell. CAP cell identification by co-localization of DAPI+ (blue), CD31+ (yellow), and EMCN+ (red) signals (Scale bar: 30 μm).


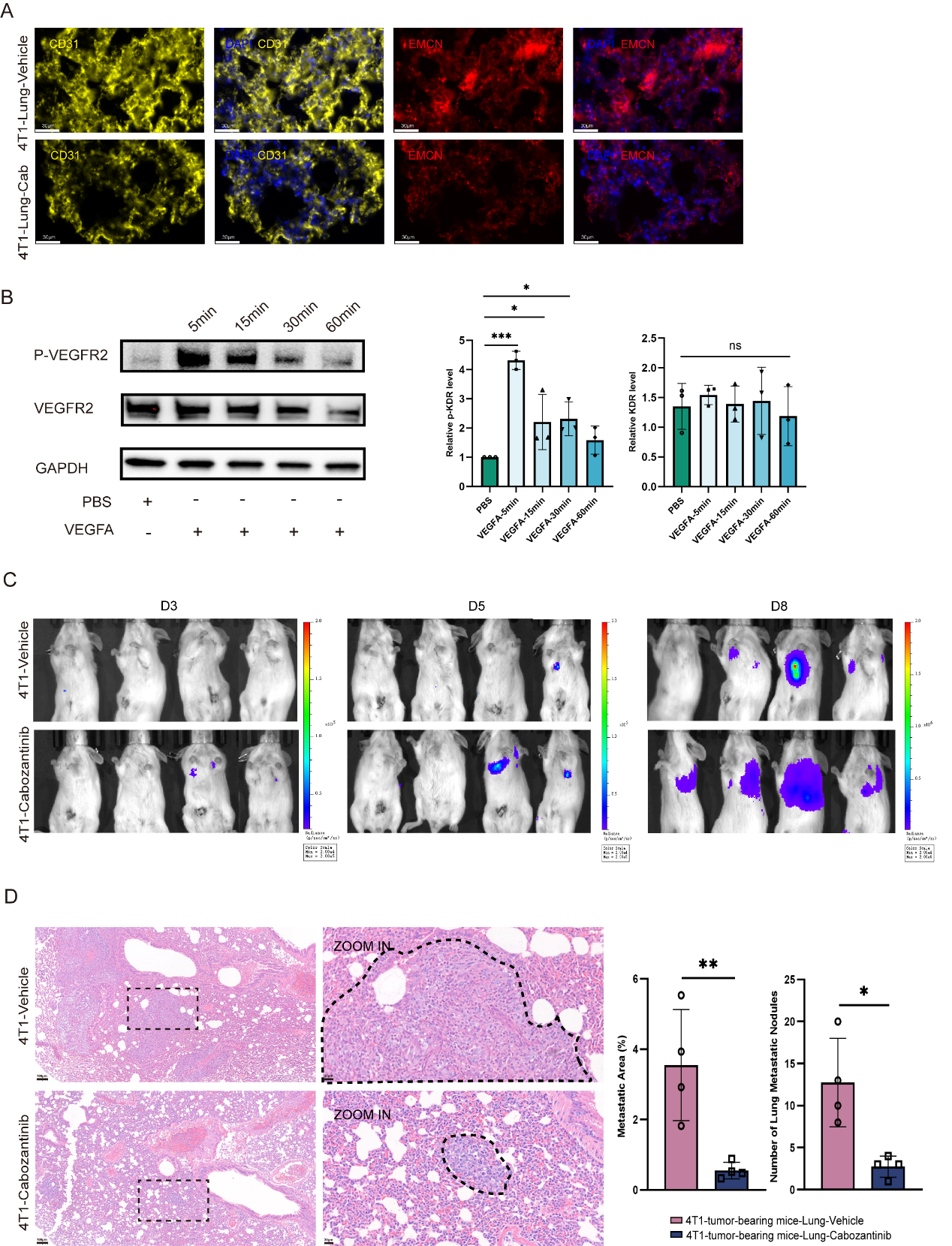


**Figure S5. Pharmacological inhibition of KDR disrupts PMN formation. (A)** Immunofluorescence analysis of PMN markers in micro-organ chip cultured lung tissues. Nuclear staining (DAPI, blue), Angiogenesis (CD31, yellow)**,** Microvasculature (EMCN, red). (Scale bars: 30 µm). **(B)** **Left:** Western blot analysis of phosphorylated VEGFR2 (p-KDR) and VEGFR2 (KDR) in protein extracts from mouse lung tissue fragments stimulated with PBS or VEGFA (100 ng/mL) for the indicated time periods (5 min to 1 h). **Right:** Quantitative analysis of phosphorylated KDR and total KDR protein levels, normalized to GAPDH. Data are presented as mean ± SD from three independent experiments (n=3). **P* < 0.05, ***P* <0.01, ****P* < 0.001 compared to the respective PBS control group at each time poi. Statistical significance was determined by one-way ANOVA; **P* <0.05, ***P* <0.01, ****P*<0.001. **(C)** Longitudinal bioluminescence imaging of pulmonary metastases at 3-8 days post-4T1-GL cells injection (n=4). **(D)** **Left:** Representative H&E-stained lung sections from vehicle- or Cabozantinib-treated 4T1 tumor-bearing mice after 15 days of treatment. Scale bars: 100 μm (overview), 20 μm (magnified views). **Right:** Quantitative analysis of lung metastasis. Metastatic burden, presented as the percentage of total lung section area occupied by metastatic regions. Number of metastatic foci per lung section. Data are shown as mean ± SD (n = 4 biologically independent mice). (**P* < 0.05, ***P* < 0.01, ****P* < 0.001 by unpaired two-tailed Student's t-test).


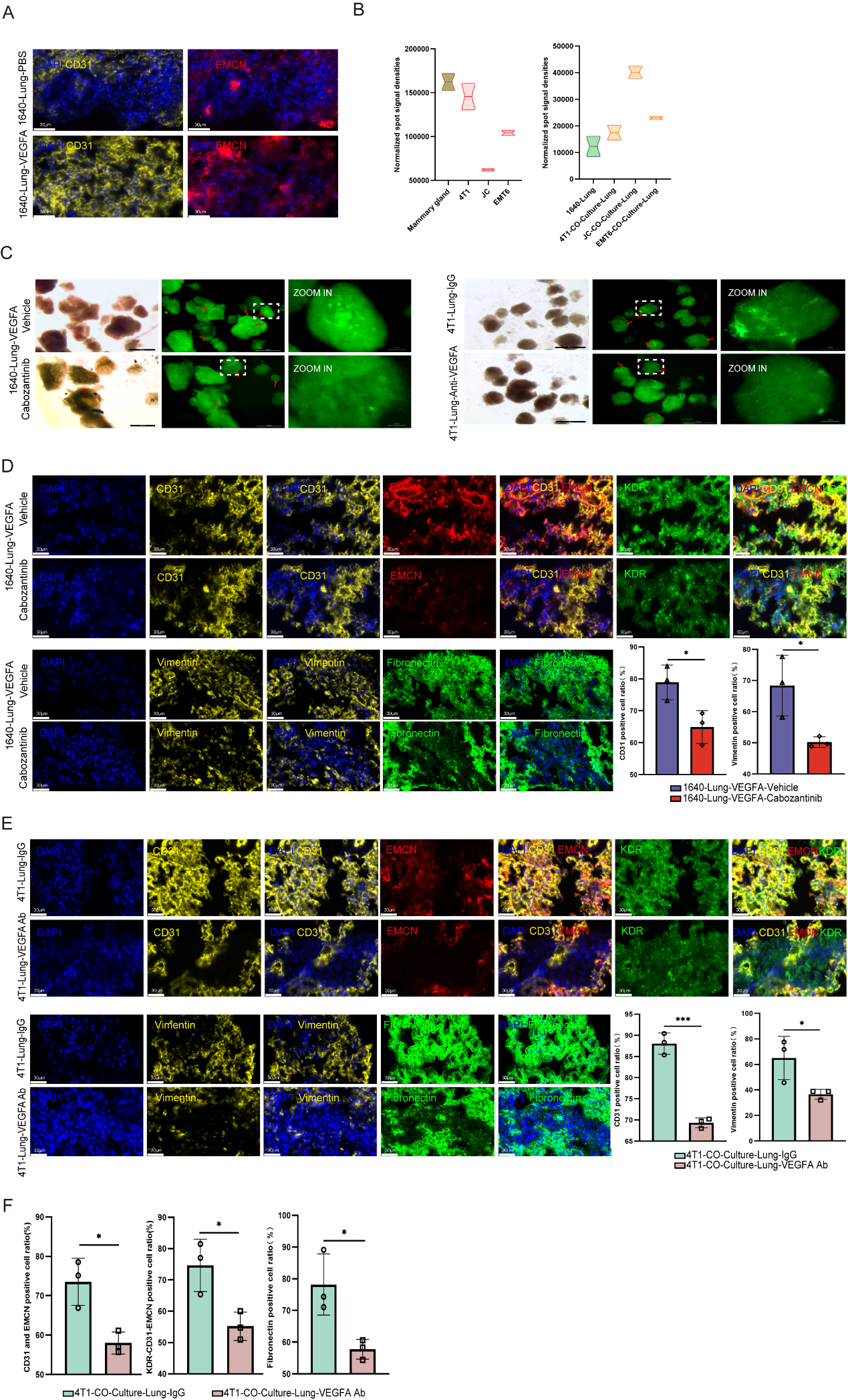


**Figure S6. VEGFA-KDR axis drives PMN formation through coordinated vascular and ECM remodeling.** **(A)** Multiplex immunofluorescence of VEGFA- versus PBS-pretreated lung tissue clusters showing: Nuclei (DAPI, blue), Angiogenesis (CD31, yellow)**,** Microvasculature (EMCN, red). (Scale bars: 30 µm). **(B)**VEGFA cytokine array dot blot analysis. **Left:** Normalized dot density profiles of tumor (4T1/EMT6/JC) versus normal mammary tissue conditioned media. **Right:** Normalized dot density profiles of tumor (4T1/EMT6/JC) co-cultured lung tissue medium versus blank culture medium (RIPM 1640) treated lung tissue supernatant. (C) **Left**: 4T1-GFP+ colonization in VEGFA protein and vehicle vs. VEGFA protein and cabozantinib groups. **Right**: 4T1-GFP+ colonization in 4T1 tumor-lung co-cultures with isotype control vs. anti-VEGFA. **(D)** **Left:** Immunofluorescence of lung tissues treated with: VEGFA+KDR inhibitor (cabozantinib) or VEGFA + vehicle control. Staining for: Angiogenesis (CD31, yellow)**,** Microvasculature (EMCN, red), KDR (green), Fibronectin (green), Vimentin (yellow), nuclei (DAPI, blue). (Scale bar: 30 μm). **Right:** Quantitative analysis of CD31+, Vimentin+ cells in VEGFA+KDR inhibitor (cabozantinib) or VEGFA + vehicle control (mean ± SEM; **P*<0.05, ***P*<0.01, ****P*<0.001 by two-tailed t-test). **(E) Left:** Immunofluorescence of 4T1 co-cultured lung tissues treated with VEGFA antibody or isotype control. Staining for: Angiogenesis (CD31, yellow)**,** Microvasculature (EMCN, red), KDR (green), Fibronectin (green), Vimentin (yellow), Nuclei (DAPI, blue). (Scale bar: 30 μm). **Right:** Quantitative analysis of CD31+, Vimentin+ cells in 4T1 co-cultured lung tissues treated with VEGFA antibody or isotype control. (mean ± SEM; **P*<0.05, ***P*<0.01, ****P*<0.001 by two-tailed t-test). **(F)** Quantification of CD31+EMCN+ (CAP cells), KDR+ fractions in CAP cells, Fibronectin deposition in 4T1 co-culture lung with isotype control or anti-VEGFA. (n=3; **P*<0.05, ***P*<0.01, ****P*<0.001 by two-tailed t-test).


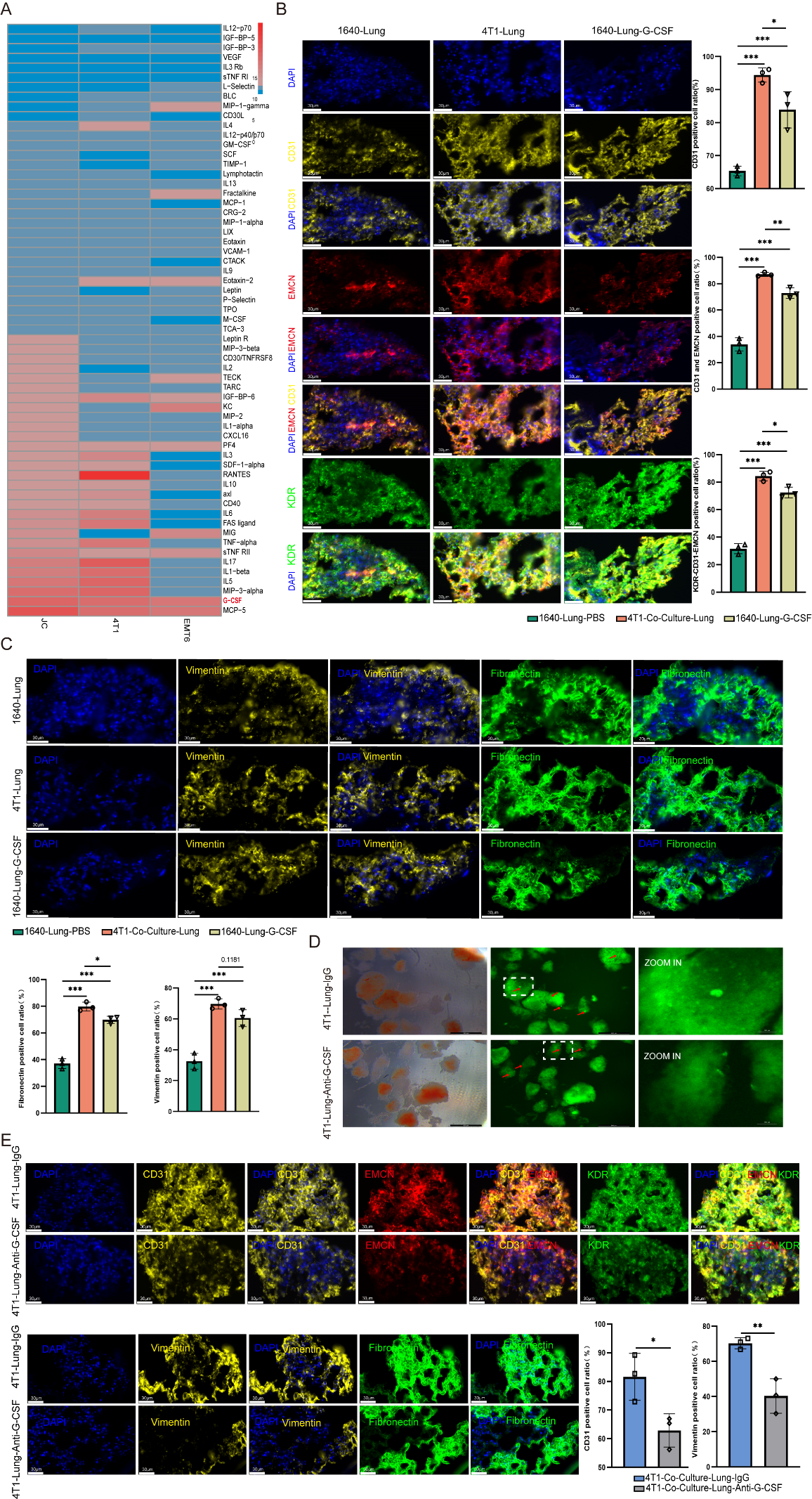


**Figure S7.** **Tumor secreted G-CSF regulated PMN** **formation through vascular and ECM remodeling. (A)** Heatmap of log10-transformed fold changes in cytokine secretion profiles comparing three distinct breast cancer subtypes (4T1, EMT6, JC) versus normal mammary gland tissue. Pink indicates upregulated cytokines in tumor supernatants, while blue denotes downregulated factors. **(B)** **Left:** Multiplex immunofluorescence analysis of angiogenesis in: G-CSF-treated, 4T1 co-cultured (positive control), RPMI 1640 (negative control) lung tissues. Markers: Angiogenesis (CD31, yellow)**,** Microvasculature (EMCN, red), nuclei (DAPI, blue), (Scale bars: 30 µm).**Right:** Quantitative assessment of CD31+, CD31+ and EMCN+, KDR+CD31+EMCN+ area fractions across treatment groups (mean ± SEM; **P*<0.05, ***P*<0.01, ****P*<0.001 by one-way ANOVA). **(C)** **Left:** Multiplex immunofluorescence analysis of ECM remodeling in: G-CSF-treated, 4T1 co-cultured (positive control), RPMI 1640 (negative control) lung tissues. Markers: Fibronectin (green), Vimentin (yellow), nuclei (DAPI, blue), (Scale bars: 30 µm).**Right:** Quantitative assessment of Fibronectin+, Vimentin+ area fractions across treatment groups (mean ± SEM; **P*<0.05, ***P*<0.01, ****P*<0.001 by one-way ANOVA). **(D)**Representative fluorescence images showing 4T1-GFP+ cell colonization in 4T1 co-cultured lung tissues treated with G-CSF antibody or isotype control. (Scale bar: 500 µm). **(E)** Immunofluorescence analysis of 4T1 co-cultured lung tissues treated with αG-CSF antibody or isotype control, showing: Vasculature (CD31+, yellow), Microvessels (EMCN+, red), KDR expression (green), Nuclei (DAPI, blue), Fibronectin (green), Vimentin (yellow), (Scale bar: 30 μm), as well as quantitative assessment of CD31+ and Vimentin+ area fractions of 4T1-preconditioned lung tissues treated with αG-CSF antibody or isotype control. (mean ± SEM; **P*<0.05, ***P*<0.01, ****P*<0.001 by two-tailed t-test).


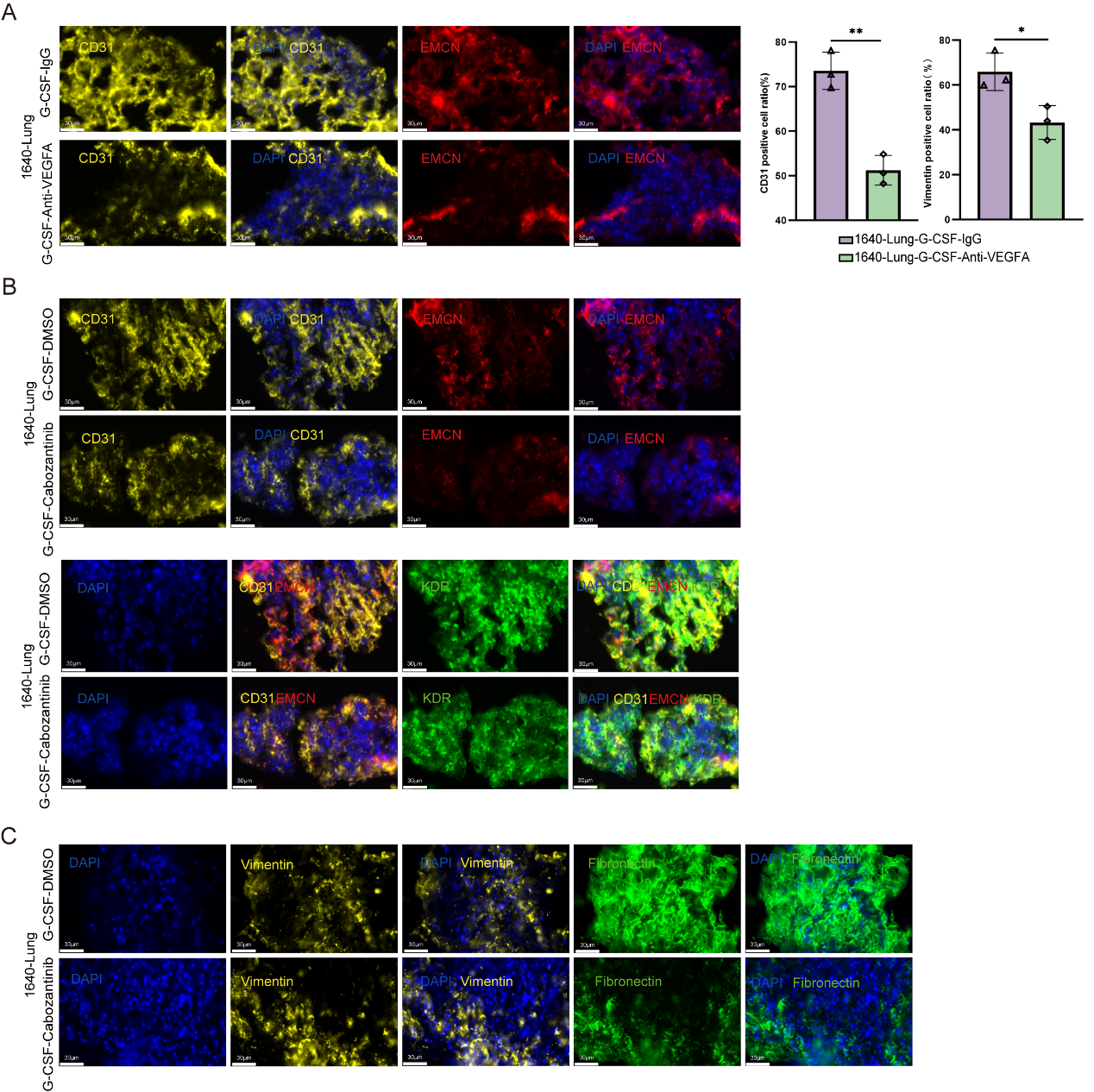


**Figure S8. Tumor-derived G-CSF regulates the VEGFA-KDR axis to promote lung PMN.** **(A)** **Left:** Multiplex immunofluorescence analysis of angiogenesis in lung tissues following G-CSF and VEGFA antibody neutralization/ isotype control: CD31+(yellow), EMCN+ (red), Nuclei (DAPI, blue), (Scale bar: 30 μm). **Right:** Quantitative assessment of CD31+ and Vimentin+ area fractions across treatment groups (mean ± SEM; **P*<0.05, ***P*<0.01, ****P*<0.001 by two-tailed t-test). **(B)** Multiplex immunofluorescence analysis of angiogenesis in lung tissues following G-CSF and cabozantinib or vehicle: CD31+(yellow), EMCN+ (red), Nuclei (DAPI, blue), KDR (green). (Scale bar: 30 μm). **(C)** Multiplex immunofluorescence analysis of ECM remodeling (Fibronectin, green; Vimentin, yellow) in lung tissues following G-CSF and cabozantinib or vehicle (Scale bars: 30 µm).
